# Preclinical animal models for onchocerciasis and loiasis: A systematic review of applications in drug screening

**DOI:** 10.1371/journal.pntd.0014401

**Published:** 2026-06-08

**Authors:** Rene Bilingwe Ayiseh, Blendin Serri Gemuh, Glory Enjong Mbah, Stephen Mbigha Ghogomu, Fidelis Cho-Ngwa

**Affiliations:** 1 Drugs and Molecular Diagnostic Laboratory (DMD), Biotechnology Unit, University of Buea, South West Region, Cameroon; 2 Department of Biochemistry and Molecular Biology (BMB), Faculty of Science, University of Buea, South West Region, Cameroon; 3 Molecular and Cell Biology Laboratory (MCBL), Biotechnology Unit, University of Buea, South West Region, Cameroon; 4 Department of Biology, Higher Teacher Training College (HTTC), The University of Bamenda, Bamenda, Cameroon; 5 Department of Chemical and Biological Engineering, National Higher Polytechnic Institute (NAHPI), The University of Bamenda, Bamenda, Cameroon; Institute of Cytology and Genetics SB RAS: FIC Institut citologii i genetiki Sibirskogo otdelenia Rossijskoj akademii nauk, RUSSIAN FEDERATION

## Abstract

**Background:**

Onchocerciasis and loiasis are co-endemic filarial neglected tropical diseases in Central and West Africa. While ivermectin-based mass drug administration has reduced onchocerciasis burden, it can trigger severe neurological adverse events in individuals with high *Loa loa* microfilaraemia. This limitation highlights the urgent need for safe macrofilaricidal therapies and appropriate preclinical models.

**Methodology/Principal findings:**

We conducted a systematic review following PRISMA guidelines to evaluate animal models used for preclinical drug screening in onchocerciasis and loiasis. Studies published between 1990 and 2025 were retrieved from PubMed and Google Scholar, and one-hundred and one eligible study were included in the qualitative synthesis. Models were assessed based on parasite stage permissiveness, survival duration, physiological relevance, host immune status, and suitability for drug evaluation. The bovine *Onchocerca ochengi* natural infection system emerged as the most physiologically relevant model, supporting the full parasite life cycle. Immunocompromised mouse models, including SCID and humanised NSG mice, allow controlled evaluation of parasite development and direct drug effects but incompletely reproduce human infection. Intraperitoneal adult male *O. ochengi* implant models in SCID mice and gerbils provide robust platforms for macrofilaricide screening. Semi-permissive rodent models offer practical systems for early-stage screening but are limited by non-physiological parasite localisation. For loiasis, non-human primate models, particularly *Papio anubis*, remain the most representative system.

**Conclusions/Significance:**

No single model fully recapitulates human co-endemic infection. While the bovine model remains the gold standard, rodent implant models enable scalable screening. The absence of a physiologically relevant co-infection model remains a major barrier to developing safe macrofilaricides.

## Author summary

Onchocerciasis (river blindness) and loiasis (African eye worm) often occur together in parts of Africa, creating a major challenge for treatment. The drug ivermectin is widely used to control onchocerciasis, but in individuals with high *Loa loa* infections it can cause severe and sometimes fatal side effects. This makes it difficult to safely treat affected populations and highlights the need for new drugs that target adult worms without harmful reactions. Animal models are essential for developing such treatments. In this review, we evaluated the main models used to study these infections and test new drugs. The cattle model using *Onchocerca ochengi* remains the most reliable system for studying infection under natural conditions. We also highlight rodent models, including mouse and gerbil implant systems, which allow controlled drug testing. However, no current model fully reproduces co-infection in humans, limiting the ability to predict drug safety. Improved models are needed to support the development of safe and effective therapies.

## 1. Introduction

Onchocerciasis (river blindness) and loiasis are filarial neglected tropical diseases (NTDs) caused by *Onchocerca volvulus* and *Loa loa*, respectively. Onchocerciasis is transmitted by blackflies of the genus *Simulium* and is characterised by dermatological and ocular pathology, including irreversible blindness. Disease manifestations are largely driven by host inflammatory responses to dying microfilariae (mf) in the skin and eyes [1–3]. Increasing evidence also links infection to neurological disorders, including epilepsy [4].

Loiasis, transmitted by *Chrysops* tabanid flies, affects an estimated 13–15 million individuals across West and Central Africa [5–7]. Clinical manifestations include Calabar swellings and migration of adult worms across subconjunctival tissues [7,8]. Chronic infections may also lead to renal, cardiac, neurological, and pulmonary complications and have been associated with excess mortality [7,9,10].

Control of onchocerciasis relies primarily on mass drug administration (MDA) with ivermectin (IVM), which effectively reduces microfilarial burden and transmission [11,12]. However, ivermectin has limited macrofilaricidal activity and requires repeated administration over prolonged periods. Critically, its use in areas co-endemic with loiasis is constrained by the risk of severe adverse neurological events, including encephalopathy and death, particularly in individuals with high *L. loa* microfilaraemia [13–16]. These safety concerns have significantly hindered elimination efforts in Central Africa [17,18].

Alternative therapeutic strategies, including anti-Wolbachia treatments such as doxycycline, have demonstrated macrofilaricidal effects through depletion of endosymbiotic bacteria [19–25]. However, prolonged treatment regimens and contraindications in vulnerable populations limit their applicability in large-scale control programmes [26]. Other candidates, including flubendazole and related compounds, have shown promise but remain constrained by formulation and safety challenges [27–30].

The development of safe and effective macrofilaricides requires robust preclinical models that accurately reflect parasite biology and drug responses. The bovine *Onchocerca ochengi* system has served as a key translational model due to its close phylogenetic relationship with *O. volvulus*, shared vector biology, and similar drug susceptibility profiles [31–37]. However, logistical and ethical constraints associated with large animal models necessitate complementary small-animal systems. Because both *O. volvulus* and *L. loa* have restricted host ranges, the development of experimental models has been challenging [38]. Existing models vary in their ability to support parasite development, mimic physiological infection, and enable evaluation of drug efficacy.

Contrary to prior reviews that have examined either *L. loa* or *Onchocerca* animal models independently [39,40], this review incorporates a translational co-endemic. Given that MDA with IVM is constrained in areas where onchocerciasis and loiasis overlap due to severe adverse neurological reactions, preclinical model evaluation must move beyond parasite permissiveness (susceptibility) alone and instead address therapeutic selectivity and safety [14,41–43].

We therefore comparatively synthesize animal models of both *Onchocerca* and *Loa* with emphasis on (i) their suitability for macrofilaricidal drug discovery, (ii) their relevance for identifying compounds effective against *Onchocerca* while avoiding rapid *L. loa* microfilaricidal effects, and (iii) their translational utility in co-endemic settings. This integrated perspective aims to bridge parasite biology with drug development to support the identification of safe and effective therapies.

## 2. Method

This review was conducted in accordance with the Preferred Reporting Items for Systematic Reviews and Meta-Analyses (PRISMA) guidelines [44]. The methodological framework was designed to enable comparative evaluation of preclinical animal models for *Onchocerca* and *Loa loa* within co-endemic settings, with emphasis on their translational relevance for drug discovery.

A comprehensive literature search was performed in PubMed and Google Scholar to identify studies published between 1990 and 2025 that investigated animal models of *O. volvulus*, *O. ochengi*, or *L. loa* infections. These databases were selected due to their extensive coverage of biomedical and parasitology literature. The search strategy used combinations of the following keywords: “animal model,” “onchocerciasis,” “loiasis,” “filarial infection,” “immunology,” and “drug screening,” with Boolean operators applied to optimise retrieval.

Studies were eligible for inclusion if they reported original data on (i) establishment or maintenance of filarial species in experimental animal hosts, (ii) host–parasite interactions or immune responses, or (iii) assessment of anti-filarial drug efficacy. Studies were excluded if they lacked original data, duplicated findings already captured elsewhere, or focused solely on human epidemiology. Review articles were screened to identify relevant primary studies but were not included in the final synthesis unless they provided unique methodological insights.

In line with the translational objectives of this review, particular emphasis was placed on models that enable evaluation of therapeutic selectivity and safety in co-endemic contexts, rather than parasite permissiveness alone. Eligible models were further assessed using predefined criteria, including parasite life-cycle stage supported, duration of parasite survival, physiological relevance of parasite localisation, host immune status, and suitability for evaluating immune-dependent versus immune-independent drug mechanisms. In addition, the level of validation of each model for drug screening—such as prior use in testing anti-Wolbachia therapies or direct-acting macrofilaricidal compounds—was considered.

A structured comparative synthesis was conducted to enable cross-parasite evaluation of *Onchocerca* and *L. loa* systems within a unified preclinical development framework. This approach facilitated identification of complementary strengths and limitations across model systems, including differences in pharmacokinetic–pharmacodynamic profiles and host immune constraints.

Emerging in vitro approaches, including organoid and co-culture systems, were identified during the screening process but were not included in the primary synthesis, as they remain insufficiently developed to recapitulate full parasite development and were outside the defined scope of preclinical animal models.

A total of 140 records were initially identified (119 from database searches and 21 from other sources). After removal of five duplicates, 135 records remained for screening. Following title and abstract screening, 113 full-text articles were assessed for eligibility. Twelve studies were excluded (five due to duplicated data and seven for being out of scope), resulting in 101 studies included in the qualitative synthesis.

**Fig 1:**
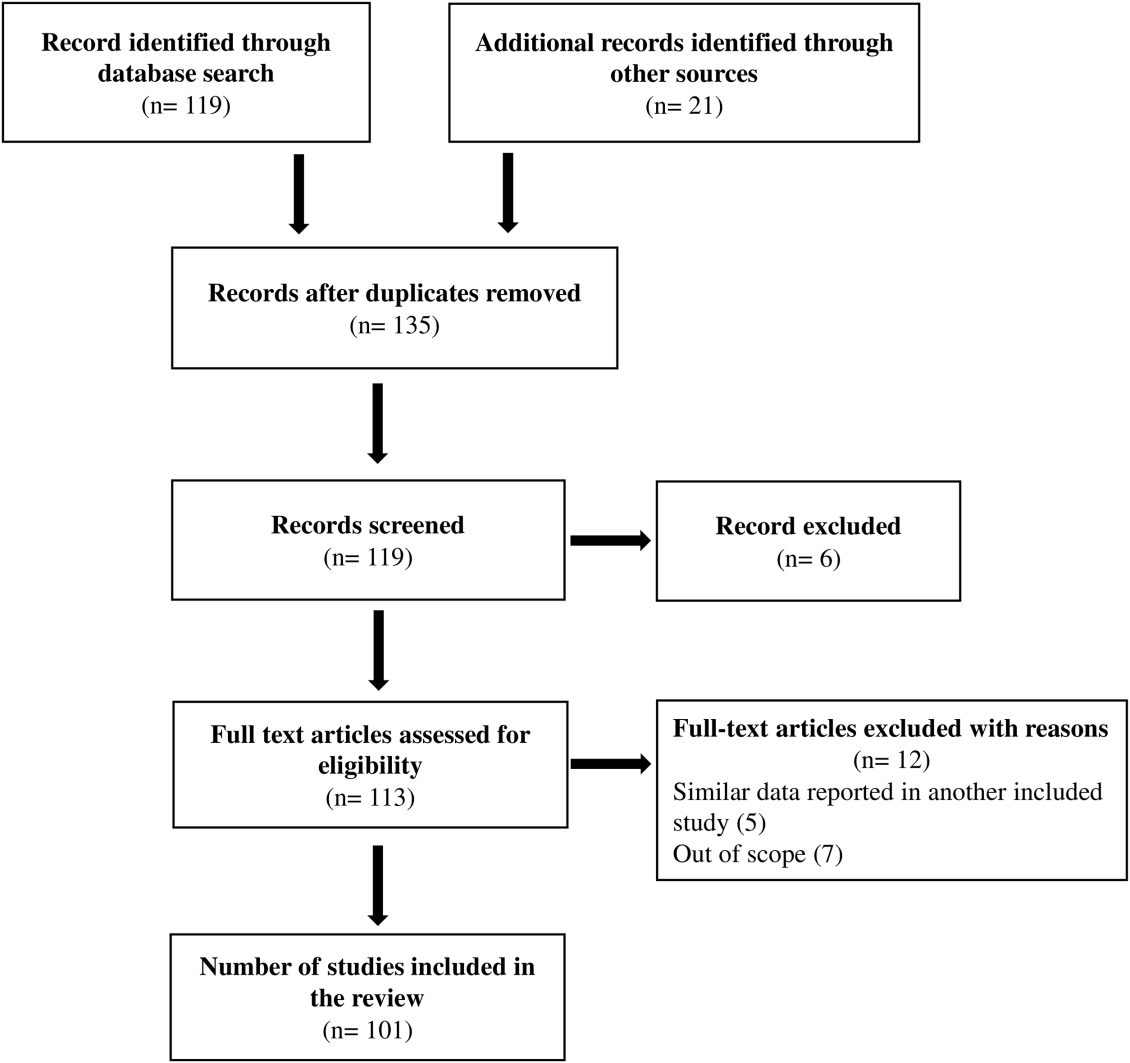
PRISMA flow diagram of study selection. A total of 140 records were identified (119 from database searches and 21 from other sources). After removing five duplicates, 135 records remained for screening, of which 119 were assessed by title and abstract. Eighty-nine full-text articles were evaluated for eligibility, and 12 were excluded (five reporting duplicate data, seven out of scope). Seventy-seven studies were finally included in this review [44].

The study selection process is summarised in the PRISMA flow diagram (Fig 1), which outlines the number of records identified, screened, and included at each stage.

## 3. Results

A total of one hundred and one [45] studies published between 1990 and 2024 met the inclusion criteria and were analysed. The selected articles collectively explored diverse animal species used to model *O. volvulus*, *O. ochengi*, and *L. loa* infections. These models provide insight into parasite biology and drug-screening potential. The findings are presented under major categories representing model types and host immunological status.

### 3.1 Onchocerciasis models

#### 3.1.1 Non-Human Primate (NHP) models of onchocerciasis.

The only susceptible animal hosts for *O. volvulus* are chimpanzees [38,46] and mangabey monkeys [47]. Chimpanzees infected with *O. volvulus* harbour patent infections that lasted between 6–9 years with adult-worm bundles located in deep tissues and mf detected 12–18 months post-infection by skin snips [48,49]. These NHP models are impractical for routine screening due to their large size, slow reproduction rate, substantial drug requirements, high cost and ethical constraints [50,51]. In contrast, *O. ochengi*, the nearest relative to *O. volvulus* [31,32,34], is more accessible, less costly to obtain, and is therefore considered a more feasible parasite model for onchocerciasis drug testing [52].

#### 3.1.2 Immunocompromised mouse models of onchocerciasis.

Immunocompetent rodents are non-permissive to *O. volvulus*, necessitating the use of immunocompromised systems such as SCID and humanised mice [53,54]. In NSG mice, *O. volvulus* L3 remained viable and developed over 4–12 weeks post-infection, with progression to advanced L4 stages and significant increases in worm size [54].

Humanised NSG mouse models of *O. volvulus* have employed distinct reconstitution strategies, including engraftment with human haematopoietic stem cells and/or immune or tissue components (CD34 + HSC “Hu-NSG”, bone-marrow–liver–thymus “BLT” mice, and human skeletal muscle cell–engrafted NSG), which variably influence parasite development [54,55]. While these models support progression from L3 to advanced L4 with development of male and female organs and an approximate 4-fold increase in larval length, parasite growth remains limited, with no evidence of fully mature adults, sexual reproduction or nodule formation *in vivo* [54,55]. These constraints highlight the partial recapitulation of human infection and the need for further optimisation of humanisation approaches and microenvironmental support to achieve complete development [54–56].

**3.1.2.1 Intraperitoneal adult worm implant model in SCID mice:** A particularly important application of immunocompromised mouse models is the intraperitoneal implantation of adult male *O. ochengi* worms in SCID mice, which has been validated as a robust macrofilaricide screening platform [53,57–59]. In this model, surgically recovered adult male worms from naturally infected cattle are implanted into the peritoneal cavity, where they remain viable and motile for approximately 5–6 weeks [53,58]. This survival window enables the assessment of drug-induced effects on worm motility, viability, and metabolic activity under controlled experimental conditions [53,57,58].

This SCID mouse implant model has been widely utilised in the preclinical evaluation of both anti-*Wolbachia* therapies and direct-acting macrofilaricidal compounds [53,58,59]. Notably, high-dose rifamycin regimens (e.g., rifapentine/rifampicin-class) and next-generation anti-Wolbachia candidates such as ABBV-4083 (flubentylosin) have demonstrated significant efficacy in depleting *Wolbachia* and impairing worm viability in filarial models, including *O. ochengi* implants and related systems [53,59,60]. In parallel, direct-acting compounds including flubendazole and related benzimidazoles or quinazoline/azaquinazoline derivatives have shown pronounced reductions in worm motility and survival in the *O. ochengi* SCID implant model and other rodent filariasis models [53,58,60,61]. These studies, particularly those led by Wanji, Turner and collaborators, have established the SCID mouse male worm implant system as a reproducible and scalable model for macrofilaricide discovery.

Although immunocompromised mouse models are limited in evaluating drugs that rely on host immune-mediated mechanisms, they provide a controlled platform for assessing direct parasite-targeting effects, as exemplified by the SCID mouse implant model [53,56,62]. Conversely, semi-permissive immunocompetent models (e.g., gerbils and hamsters), where parasites are subject to host immune pressure, may introduce confounding effects that can overestimate macrofilaricidal efficacy [63–66]. Thus, both systems present complementary advantages and limitations, and should be interpreted within the context of their immunological constraints.

Certain filaricidal interventions, particularly anti-Wolbachia therapies and immune-modulating compounds, rely in part on host immune effector mechanisms to achieve optimal efficacy [63,67,68]. Consequently, immunocompromised models may underestimate the activity of such agents, as key immune pathways involved in parasite clearance are impaired [53,63,68]. This limitation should be considered when interpreting drug efficacy data generated from these systems.

#### 3.1.3 Immunocompetent mouse model of onchocerciasis.

BALB/c mice are a short-term model for *O. ochengi* mf, as they fail to maintain the parasites for extended periods, with detectable mf typically present only within the first two weeks post-infection [65,69]. IVM effectively clears *O. ochengi* mf in this model, validating its utility for microfilaricidal studies. However, its short duration limits its suitability for evaluating slow-acting drugs, and its small size restricts its capacity to harbour large numbers of adult worms.

#### 3.1.4 Gerbil model of onchocerciasis.

Gerbils have been shown to harbour both adult and microfilarial stages of *O. ochengi* and are widely used for drug testing [58,64]. Adult male worms can survive for extended periods following implantation, while female worms are generally viable only for short durations [66]. In untreated animals, substantial recovery rates with preserved motility have been reported, whereas flubendazole (FBZ) treatment markedly reduces worm viability and motility [64,70].

Gerbils also support microfilarial infections, although recovery rates remain relatively low. IVM effectively clears *O. ochengi* mf in this model, confirming its suitability for microfilaricidal evaluation [65]. Overall, the gerbil model is advantageous due to its ability to support both short-term female worm survival and longer-term male worm persistence, while being sufficiently large to accommodate multiple adult parasites.

**3.1.4.1 Intraperitoneal adult male implant model in gerbils:** In addition to their role in general drug screening, gerbils have been extensively used in adult male *O. ochengi* intraperitoneal implant models, which complement SCID mouse systems and provide an alternative platform for macrofilaricide evaluation [64,70]). In this model, adult male worms are implanted into the peritoneal cavity of Mongolian gerbils, where they can survive for extended periods of up to 35–42 days with substantial recovery rates and preserved motility in untreated controls [64,70].

The gerbil implant model has been particularly valuable in evaluating anti-*Wolbachia* therapies, including flubentylosin (ABBV-4083), where drug efficacy is assessed through reductions in *Wolbachia* load and subsequent impairment of worm viability [71–74]. Importantly, this model has also been used within broader rodent systems to assess pharmacokinetic–pharmacodynamic relationships across species, highlighting differences in drug exposure and efficacy between rodents [71–74]. As such, the gerbil system provides complementary insights to SCID mouse models and strengthens confidence in translational drug candidates [64,71–73].Notably, the gerbil *O. ochengi* adult male implant model has also been applied in anti-Wolbachia drug screening, including flubentylosin (ABBV-4083), where reductions in Wolbachia burden correlate with impaired worm viability [59,71,73]. Importantly, comparative use of this model alongside SCID mouse systems has highlighted species-specific pharmacokinetic differences that influence drug exposure and efficacy, underscoring the value of employing multiple complementary models in macrofilaricide development [58,59,73,75].

#### 3.1.5 Hamster model of onchocerciasis.

The Syrian hamster has proven to be a useful model for both microfilarial and adult stages of *O. ochengi*. Microfilariae can be detected up to 30 days post-infection and are effectively cleared following IVM treatment [70]. Hamsters can also support adult female worms beyond 30 days post-implantation, although recovery rates remain low [66]. Despite these limitations, the hamster model provides a practical system for evaluating both microfilaricidal and macrofilaricidal drug candidates.

Summary of all the different animal models of onchocerciasis, including worm stages, key findings, limitations, and drug use for validation, is presented in Table 1.

**Table 1:** Summary of animal models for onchocerciasis, worm stage used, key findings, limitations and drug use for validation.

S/N	Model	Worm Stage	Key Findings	Limitations	Drug use for validation
1	Cattle (*O. ochengi*)	Full life cycle (L3 → adult → mf)	Gold-standard natural infection model; supports full parasite development, nodule formation, and longitudinal drug evaluation [23,33,76,52]	Expensive; logistically demanding; requires specialized infrastructure	Tetracyclines, flubendazole, ivermectin, emodepside, anti-Wolbachia drugs
2	SCID mice (adult worm implant model)	Adult male *O. ochengi*	Worm survival ~5–6 weeks post-implantation; robust and reproducible macrofilaricide screening platform [53,57–59]	Immune-deficient; lacks host immune contribution	Anti-Wolbachia (rifampicin, flubentylosin); macrofilaricides (flubendazole, quinazolines)
3	NSG/ humanised mice	L3 → advanced L4	Supports partial development of *O. volvulus* larvae with organ differentiation [54,55]	No full maturation, reproduction, or nodule formation; incomplete recapitulation of human infection	IVM
4	BALB/c mice	Microfilaria (*O. ochengi*)	Short-term mf survival; suitable for evaluating microfilaricidal activity [65,69]	Short duration; not suitable for slow-acting drugs; limited capacity for adult worms	IVM
5	Gerbils (general infection model)	Adult + mf (*O. ochengi*)	Supports short-term female worms and longer survival of male worms; suitable for micro- and macrofilaricidal screening [58,64,66,70]	Female worms short-lived; non-physiological parasite localisation	IVM; flubendazole
6	Gerbils (implant model)	Adult male *O. ochengi*	Worm survival up to ~35–42 days; enables PK/PD evaluation and cross-species comparison [64,70]	Variable recovery; non-natural parasite location	Anti-Wolbachia (flubentylosin); macrofilaricides
7	Hamsters	Microfilaria + adult female *O. ochengi*	Supports mf and adult female worms; suitable for combined micro- and macrofilaricidal evaluation [66,69]	Low recovery rates; limited long-term survival	IVM; flubendazole
8	NHPs (chimpanzee, mangabey)	Full life cycle (*O. volvulus*)	Closest model to human infection; long-term parasite survival and natural tissue localisation [38,46–49]	Ethical constraints; high cost; impractical for routine screening	Limited experimental use

FBZ = flubendazole, IVM = ivermectin, L3 = third-stage larvae, Mf = microfilaria, NHPs = non-human primates.

Recent studies showed that cattle-related factors affect the survival of male *O. ochengi* worms in mice and gerbils [57]. The survival of *O. ochengi* mf in experimental animals has been observed to differ across parasite batches.

### 3.2 Loiasis models

#### 3.2.1 Non-human primate models of loiasis.

Aside from humans, only non-human primates (NHPs) have been shown to be highly susceptible to *L. loa* [77]. One of the most studied systems is the baboon model, which has been instrumental in understanding parasite biology and drug responses [78–81].

Importantly, the baboon non-human primate (NHP) model of loiasis has been further refined by Wanji and McKenzie and colleagues, who demonstrated its utility in investigating ivermectin-associated adverse events [82–84]. In this model, hyper-microfilaraemic *Papio anubis* reproduced key clinical, hematological, and neuropathological features observed in humans following ivermectin treatment, including rapid microfilarial clearance and associated inflammatory responses [82,83,85]. These studies have provided critical insights into the pathogenesis of severe adverse events and remain highly relevant for evaluating the safety of antifilarial interventions in *L. loa*-endemic settings [82–84,86].

Although NHP models closely reproduce human infection, their use is constrained by ethical considerations, high costs, and logistical complexity. As a result, alternative rodent models have been developed for scalable experimental studies.

#### 3.2.2 Immunocompromised mouse models of loiasis.

Immunocompromised mouse models have played a central role in advancing the study of *L. loa* by overcoming the natural resistance of immunocompetent rodents to filarial infection. A wide range of genetically modified and lymphopenic mouse strains have been employed to support parasite development, enabling controlled investigation of host–parasite interactions and drug responses.

In mice with disrupted Th2 cytokine signaling, *L. loa* L3 larvae are able to progress beyond early developmental stages, and in lymphopenic mice they can mature into adult worms capable of producing microfilariae [87]. These findings highlight the critical role of host immune responses—particularly type-2 immunity—in restricting parasite development.

Several targeted immune-deficient models have been used to further dissect these interactions. CCR-3 knockout mice, which are deficient in eosinophil recruitment, exhibit prolonged larval survival, demonstrating the importance of eosinophils in early parasite clearance [88]. Similarly, cytokine-deficient strains lacking IL-4R, IL-5, or related signaling pathways support extended survival and development of *L. loa* larvae into immature or young adult stages [87,89]. Across these models, parasites have been recovered from multiple anatomical sites, including subcutaneous tissues, muscles, and cardiopulmonary compartments, reflecting a broad tissue distribution consistent with migratory larval behaviour.

More advanced immunodeficient strains have enabled sustained infections that more closely approximate aspects of human loiasis. While some strains such as CB.17 SCID mice support only limited parasite development, others—including compound immunodeficient models such as NOD.SCIDγc ⁻ /⁻ and BALB/c RAG2 ⁻ / ⁻ γc ⁻ / ⁻ mice—permit the development of fertile adult worms and the establishment of microfilaraemia [84,89]. In these models, adult worms localize primarily within subcutaneous tissues and muscle fascia, with additional presence in cardiopulmonary and serosal compartments. This distribution supports the hypothesis that *L. loa* undergoes a lymphatic migratory phase during development.

Implantation studies have further demonstrated that adult *L. loa* worms can establish stable infections in immunodeficient hosts, leading to sustained microfilaraemia [84]. These approaches provide a valuable platform for studying parasite reproduction and drug effects under controlled conditions.

Comparative infection studies have also shown that these models exhibit a high degree of specificity for *L. loa*, with limited or no successful establishment of other human filarial species under similar conditions [56]. This reinforces their suitability for focused investigations of loiasis.

Overall, immunocompromised mouse models—including SCID, NOD SCID, CCR-3 knockout, cytokine-deficient strains, and RAG2/γc-deficient mice—provide powerful tools for studying *L. loa* biology and evaluating antifilarial drugs. Their capacity to support prolonged parasite survival makes them particularly useful for assessing slow-acting therapies. However, the absence of functional immune responses remains a key limitation, as it restricts evaluation of interventions that depend on host immune-mediated mechanisms.

#### 3.2.3 Immunocompetent mouse models of loiasis.

In contrast to immunocompromised systems, immunocompetent mouse models are inherently restrictive to *L. loa* infection due to intact host immune responses. While these models do not support sustained parasite development or reproduction, they provide valuable insight into early host–parasite interactions and are particularly useful for evaluating rapid-acting microfilaricidal interventions.

Following experimental infection, *L. loa* microfilariae are detectable only transiently in immunocompetent mice, reflecting rapid immune-mediated clearance. In these mouse models, circulating *L. loa* microfilariae are typically scant and transient in peripheral blood, with the majority sequestered within the cardiopulmonary system, particularly the heart and lungs [84,87]. Despite this non-physiological distribution, IVM and other microfilaricidal agents have consistently demonstrated effective clearance of microfilariae from these compartments, supporting the utility of this model for evaluating microfilaricidal activity against *L. loa* [65,84,87]. Unlike immunocompromised models, where parasite survival is prolonged due to impaired immune function, immunocompetent systems impose strong immunological pressure that limits parasite persistence and prevents development to adult stages. This fundamental difference restricts their application for studying long-term infection dynamics or evaluating macrofilaricidal compounds. However, it also provides a more physiologically relevant context for assessing immune-mediated parasite clearance mechanisms.

The rapid decline of microfilariae in these models is largely attributed to effective innate and adaptive immune responses, including eosinophil-mediated cytotoxicity and cytokine-driven clearance pathways. As such, these models are well suited for investigating early-stage infection dynamics and host immune responses but are less appropriate for studies requiring prolonged parasite survival.

From a drug development perspective, immunocompetent mouse models are most informative for evaluating fast-acting microfilaricidal agents, where short-term parasite clearance is the primary endpoint. In contrast, they are poorly suited for assessing slow-acting or immune-independent macrofilaricidal compounds, which require sustained parasite viability over extended periods.

Taken together, immunocompetent and immunocompromised mouse models represent complementary experimental systems. While immunocompromised models enable extended parasite survival and evaluation of direct drug effects, immunocompetent models provide insight into immune-mediated clearance and early host–parasite interactions. The choice between these systems should therefore be guided by the specific biological question and the mechanism of action of the therapeutic candidate under investigation.

#### 3.2.4 Gerbil model of loiasis.

Gerbils have been shown to be more permissive to *L. loa* infection than mice, supporting longer persistence of microfilariae following experimental infection [65]. However, microfilariae in this model are largely confined to the peritoneal cavity rather than circulating in the bloodstream, limiting physiological relevance to human infection.

Despite this limitation, the gerbil model provides a practical system for evaluating drug effects on parasite survival under controlled conditions.

Table 2 summarizes all the different animal models of loiasis, showing the different worm stages, key findings and limitations.

**Table 2:** Summary of animal models for loiasis, worm stage used, key findings, limitations and drug use for validation.

S/N	Model	Worm Stage	Key Findings	Limitations	Drug use for validation
1	*Papio anubis* (baboon)	L3 → mf	Reproduces ivermectin-associated adverse events; models hyper-microfilaraemia and neuropathology [82,83]	High cost; ethical constraints	IVM
2	CB.17 SCID mice	mf	Rapid clearance of mf following IVM; useful for microfilaricidal studies [84]	Limited immune function; partial parasite development	IVM
3	NOD SCID mice	L3	Limited support for parasite development; infection often cleared	Poor long-term maintenance	–
4	NOD.SCIDγc − /−	L3 → adult → mf	Supports full development to fertile adults and sustained infection [84,89]	Immune-deficient; lacks physiological immune response	IVM
5	BALB/c RAG2 − /−	L3	Partial parasite development; supports implantation models [84]	Limited immune context	IVM
6	BALB/c RAG2 − / − γc − /−	L3 → adult → mf	Sustained parasite survival and microfilaraemia; suitable for long-term studies [84,89]	Immune-deficient	IVM
7	Cytokine-deficient mice (IL-4R, IL-5, IFN-γ, CCR-3 KO)	L3 → immature adult	Extended larval survival; highlights role of Th2 and eosinophils in parasite clearance [87–89]	Do not fully reproduce infection	–
8	WT BALB/c mice	mf	Transient mf presence; sequestration in cardiopulmonary system [84,87]	Short-lived infection	IVM
9	Gerbils	mf	Higher permissiveness than mice [65]	Mf localized in peritoneum	IVM

IVM= ivermectin, L3= third-stage larvae, Mf= microfilaria.

### 3.3 Co-infection model

A co-infection model combining *L. loa* and *O. ochengi* has been developed in gerbils, enabling simultaneous assessment of both parasites within a single host system [65]. In this model, IVM effectively clears *O. ochengi* microfilariae and reduces *L. loa* motility.

However, the model remains limited by non-physiological parasite localisation and does not fully reproduce the dynamics of co-endemic human infections. Nonetheless, it represents an important step toward developing experimental platforms capable of evaluating drug safety and efficacy in co-infection contexts.

## 4. Discussion

These findings highlight important trade-offs between physiological relevance, immune competence, and scalability across available models.

### 4.1 Overview of current animal models in a translational context

The present review synthesizes available animal models for onchocerciasis and loiasis within a translational framework centered on drug discovery in co-endemic settings. Across model systems, a recurring theme is that no single model fully recapitulates human infection, particularly in terms of parasite development, physiological localization, and host immune competence. Instead, each model captures specific aspects of parasite biology and drug response, necessitating a complementary, multi-model approach.

The bovine *O. ochengi* natural infection system remains the most physiologically relevant model, supporting the complete parasite life cycle under natural transmission conditions and enabling longitudinal assessment of macrofilaricidal efficacy [23,33,52]. However, its operational complexity and cost limit its use to late-stage validation. In contrast, small-animal models—particularly rodents—provide scalable platforms for early-stage screening, albeit with important biological constraints.

### 4.2 Strengths and limitations of onchocerciasis models

#### 4.2.1 Natural and large-animal models.

The bovine *O. ochengi* natural infection model represents the most physiologically relevant and widely validated surrogate system for human onchocerciasis. As the closest phylogenetic relative of *O. volvulus*, *O. ochengi* shares similar transmission vectors, nodule formation, Wolbachia endosymbiosis, and drug susceptibility profiles, making it a cornerstone model for macrofilaricide discovery [33,52].

Over the past three decades, this model has been extensively utilised in both experimental and field settings, particularly in Cameroon, to evaluate candidate macrofilaricides and anti-Wolbachia therapies [23,33,76,90] including recent translational drug candidates [59]. Notably, tetracycline-class antibiotics demonstrated potent macrofilaricidal activity through depletion of Wolbachia, leading to worm sterility and death [91]. Similarly, modified flubendazole formulations showed significant reductions in worm motility and viability, while long-acting IVM achieved sustained microfilarial clearance [76,92]. More recently, emodepside has emerged as a promising slow-acting macrofilaricide, inducing paralysis, sterility, and eventual death of adult worms [93].

A major strength of the bovine model lies in its ability to support the full parasite life cycle under natural infection conditions, enabling longitudinal assessment of drug efficacy on adult worms, nodules, and microfilariae. This provides a level of translational relevance that cannot be achieved in small animal models. However, despite its robustness, the model is constrained by high operational costs, logistical complexity, and ethical considerations associated with large animal use. These limitations have driven the development of complementary rodent models for early-stage drug screening. Nevertheless, the bovine *O. ochengi* system remains the gold standard for validating macrofilaricidal candidates prior to clinical development.

This model’s unparalleled translational relevance is due to its close phylogenetic relationship with *O. volvulus*, shared vector biology, and validated responses to key drug classes [23,33]. Yet, its cost and complexity restrict routine use, underscoring the importance of scalable rodent models for initial screening.

#### 4.2.2 Immunocompromised rodent models.

Immunodeficient mouse models (e.g., SCID, NSG) have significantly advanced the study of *O. volvulus* by permitting partial parasite development and supporting adult worm survival following implantation [53,58]. These systems provide highly controlled platforms for evaluating direct parasite-targeting drugs, including anti-Wolbachia compounds such as flubentylosin and rifamycin derivatives [59,94]. However, their major limitation is the absence of functional immune responses, which restricts their ability to evaluate therapies that rely on host immune mechanisms [67,63]. Furthermore, humanized NSG models, although capable of supporting larval development to advanced stages, fail to achieve full parasite maturation, sexual reproduction, or nodule formation, reflecting only partial recapitulation of human infection [54–56].

#### 4.2.3 Semi-permissive rodent models (Gerbils and hamsters).

Gerbils and hamsters provide practical and scalable models for drug screening, supporting short- to medium-term survival of *O. ochengi* microfilariae and adult worms [64–66,69]. Notably, adult male worm implant models in gerbils and SCID mice have been widely used for macrofilaricide evaluation, with reproducible worm recovery and viability over several weeks [53,58,64,69].

Despite these advantages, these models are constrained by non-physiological parasite localization, typically within the peritoneal cavity, and by variable parasite recovery rates. In addition, host immune pressure in immunocompetent rodents may introduce confounding effects that could overestimate drug efficacy [65,63]. These limitations underscore the need for cautious interpretation of efficacy data derived from such systems.

### 4.3 Strengths and limitations of loiasis models

#### 4.3.1 Non-human primate models.

Non-human primates, particularly baboons, represent the most biologically accurate models of *L. loa* infection, closely reproducing human microfilaraemia dynamics and clinical responses [82,83]. These models have been critical in elucidating the pathogenesis of IVM-associated severe adverse events, including rapid microfilarial clearance and associated inflammatory responses.

However, their application is limited by ethical constraints, high costs, and restricted availability, rendering them unsuitable for routine drug screening.

#### 4.3.2 Immunocompromised mouse models.

Immunodeficient mouse models have emerged as valuable tools for studying *L. loa*, particularly for assessing microfilaricidal activity and adult worm development [84,87]. These include SCID, NOD SCID, RAG2 ⁻ / ⁻ , RAG2 ⁻ / ⁻ γc ⁻ / ⁻ , and cytokine-deficient strains, which collectively enable varying degrees of parasite development and survival. These models demonstrate that while peripheral microfilaraemia is often low and transient, microfilariae predominantly sequester in cardiopulmonary tissues, where they remain accessible to drug action.

Despite this non-physiological distribution, these systems have been validated for evaluating microfilaricidal efficacy, as demonstrated by consistent IVM-mediated clearance [84,87]. Nevertheless, similar to onchocerciasis models, their lack of immune competence limits their utility for studying immune-mediated mechanisms.

### 4.4 Translational gaps and limitations across models

A key finding from this review is the absence of a unified model that simultaneously satisfies three critical criteria:

1. Physiological parasite localization
2. Full parasite development and longevity
3. Intact host immune competence

Current models typically fulfill only one or two of these criteria. For example:

- The bovine model achieves physiological relevance but lacks scalability
- Immunocompromised rodents enable parasite survival but lack immune context
- Semi-permissive rodents offer practicality but compromise on localization and consistency

These limitations are particularly critical in the context of co-endemic onchocerciasis–loiasis, where drug safety and selectivity must be evaluated simultaneously.

### 4.5 Future directions and model innovation

Addressing these gaps will require the development of next-generation integrative platforms. Humanized mouse models represent a promising direction, particularly if further refined to support:

- Complete parasite maturation
- Physiological tissue localization
- Functional immune interactions

Emerging *in vitro* approaches, including organoid systems and advanced co-culture models, are beginning to provide complementary platforms for filarial research [95–97]. Although these systems remain underdeveloped for fully recapitulating parasite development and host–parasite interactions, recent studies highlight their potential for early-stage drug screening and mechanistic investigations [63,95,97,98]. Integration of such platforms with established animal models may enhance translational pipelines and reduce reliance on *in vivo* systems [63,95,99,45].

In addition, the development of co-infection models that can simultaneously accommodate *Onchocerca* and *L. loa* parasites would significantly enhance the predictive value of preclinical studies, particularly for identifying macrofilaricidal compounds that avoid rapid *L. loa* microfilarial clearance.

Finally, a multi-model pipeline approach, integrating high-throughput rodent screening with validation in physiologically relevant systems such as the bovine model, may provide the most practical pathway for accelerating safe and effective drug discovery.

### 4.6 Conclusion

In summary, while substantial progress has been made in developing animal models for onchocerciasis and loiasis, each system presents distinct advantages and limitations. A strategic combination of these models, coupled with continued innovation in humanized and co-infection platforms, will be essential to overcome current translational barriers and to advance the development of safe macrofilaricidal therapies in co-endemic regions.

## Supporting information

S1 Fig PRISMA flow diagram of study selection. A total of 140 records were identified (119 from database searches and 21 from other sources). After removing five duplicates, 135 records remained for screening, of which 119 were assessed by title and abstract. Eighty-nine full-text articles were evaluated for eligibility, and 12 were excluded (five reporting duplicate data, seven out of scope). Seventy-seven studies were finally included in this review.
(PDF)

S1 Table Summary of animal models for onchocerciasis, worm stage used, key findings, limitations and drug use for validation. Table list 8 animal models that have been developed for onchocerciasis, the different stages used and the drugs used to validate the models.
(PDF)

S2 Table Summary of animal models for loiasis, worm stage used, key findings, limitations and drug use for validation. Table list 9 animal models that have been developed for loiasis, the different stages used and the drugs used to validate the models.
(PDF)

S1 Checklist Prisma Checklist. (DOCX)
