# Supplementary material for: Preclinical animal models for onchocerciasis and loiasis: A systematic review of applications in drug screening
Source: PLoS Negl Trop Dis. 2026 Jun 8;20(6):e0014401. doi: 10.1371/journal.pntd.0014401 (PMC13271504; doi:10.1371/journal.pntd.0014401)
Supplement: S1 Fig — A total of 140 records were identified (119 from database searches and 21 from other sources). After removing five duplicates, 135 records remained for screening, of which 119 were assessed by title and abstract. Eighty-nine full-text articles were evaluated for eligibility, and 12 were excluded (five reporting duplicate data, seven out of scope). Seventy-seven studies were finally included in this review. (PDF) [file pntd.0014401.s001.pdf]

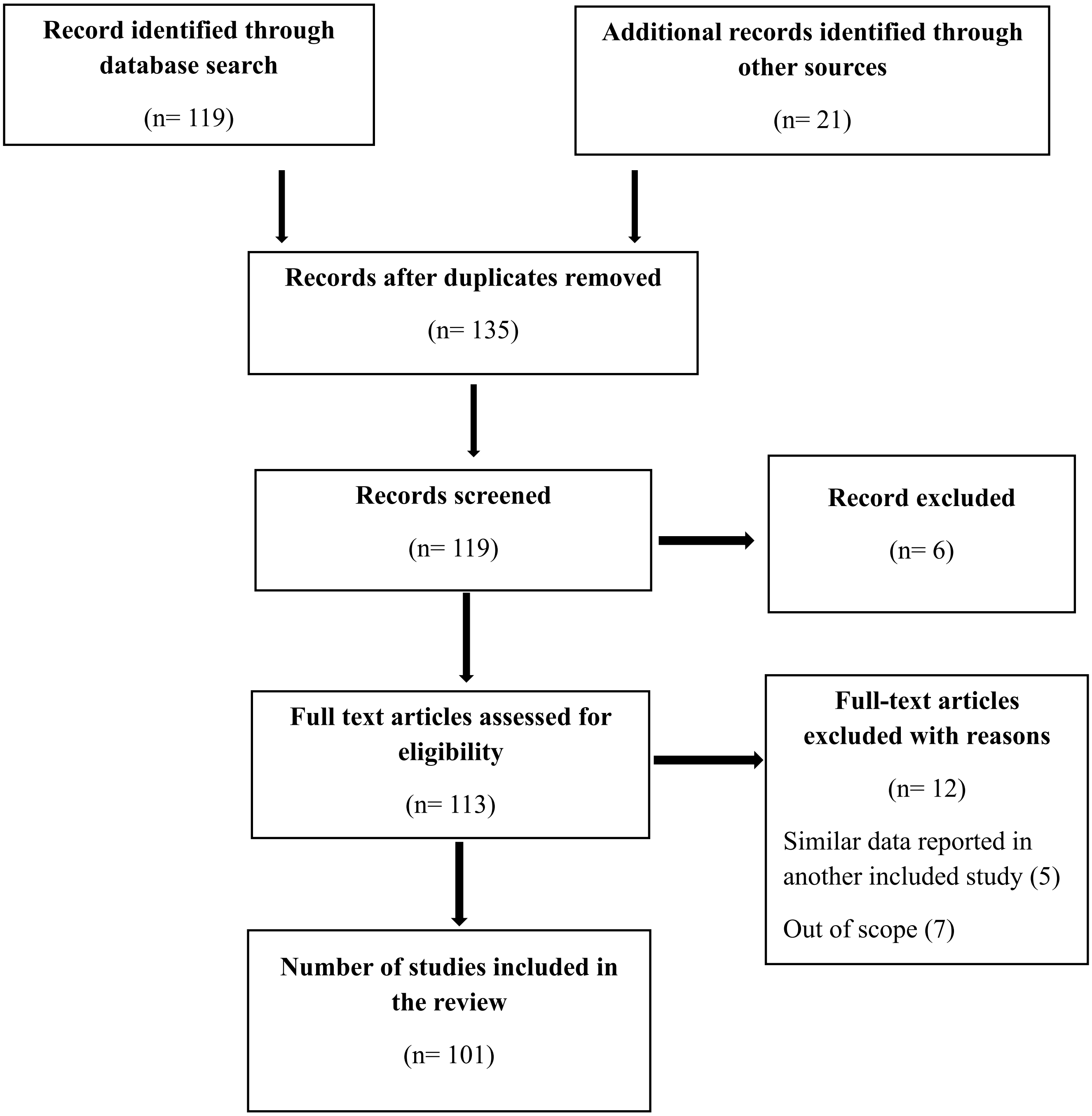


**Fig 1. PRISMA flow diagram of study selection.** A total of 140 records were identified (119 from database searches and 21 from other sources). After removing five duplicates, 135 records remained for screening, of which 119 were assessed by title and abstract. Eighty-nine full-text articles were evaluated for eligibility, and 12 were excluded (five reporting duplicate data, seven out of scope). Seventy-seven studies were finally included in this review
