# Supplementary material for: Preclinical animal models for onchocerciasis and loiasis: A systematic review of applications in drug screening
Source: PLoS Negl Trop Dis. 2026 Jun 8;20(6):e0014401. doi: 10.1371/journal.pntd.0014401 (PMC13271504; doi:10.1371/journal.pntd.0014401)
Supplement: S1 Table — Table list 8 animal models that have been developed for onchocerciasis, the different stages used and the drugs used to validate the models. (PDF) [file pntd.0014401.s002.pdf]

Table 1. Summary of animal models for onchocerciasis, worm stage used, key findings, limitations and drug use for validation.

S/N	Model	Worm Stage	Key Findings	Limitations	Drug use for validation
1	Cattle (*O. ochengi*)	Full life cycle (L3 → adult → mf)	Gold-standard natural infection model; supports full parasite development, nodule formation, and longitudinal drug evaluation (23,33,75,76)	Expensive; logistically demanding; requires specialized infrastructure	Tetracyclines, flubendazole, ivermectin, emodepside, anti-Wolbachia drugs
2	SCID mice (adult worm implant model)	Adult male *O. ochengi*	Worm survival ~5–6 weeks post-implantation; robust and reproducible macrofilaricide screening platform (52,56–58)	Immune-deficient; lacks host immune contribution	Anti-Wolbachia (rifampicin, flubentylosin); macrofilaricides (flubendazole, quinazolines)
3	NSG / humanised mice	L3 → advanced L4	Supports partial development of *O. volvulus* larvae with organ differentiation (53,54)	No full maturation, reproduction, or nodule formation;	IVM

				incomplete recapitulation of human infection	
4	BALB/c mice	Microfilaria (*O. ochengi*)	Short-term mf survival; suitable for evaluating microfilaricidal activity (64,68)	Short duration; not suitable for slow-acting drugs; limited capacity for adult worms	IVM
5	Gerbils (general infection model)	Adult + mf (*O. ochengi*)	Supports short-term female worms and longer survival of male worms; suitable for micro- and macrofilaricidal screening (57,63,65,69)	Female worms short-lived; non-physiological parasite localisation	IVM; flubendazole
6	Gerbils (implant model)	Adult male *O. ochengi*	Worm survival up to ~35–42 days; enables PK/PD evaluation and	Variable recovery; non-natural	Anti-Wolbachia (flubentylosin); macrofilaricides

			cross-species comparison (63,69)	parasite location	
7	Hamsters	Microfilaria + adult female *O. ochengi*	Supports mf and adult female worms; suitable for combined micro- and macrofilaricidal evaluation (65,68)	Low recovery rates; limited long-term survival	IVM; flubendazole
8	NHPs (chimpanzee, mangabey)	Full life cycle (*O. volvulus*)	Closest model to human infection; long-term parasite survival and natural tissue localisation (38,45–48)	Ethical constraints; high cost; impractical for routine screening	Limited experimental use

FBZ= flubendazole, IVM= ivermectin, L3= third-stage larvae, Mf= microfilaria, NHPs= non-human primates
