# Supplementary material for: Preclinical animal models for onchocerciasis and loiasis: A systematic review of applications in drug screening
Source: PLoS Negl Trop Dis. 2026 Jun 8;20(6):e0014401. doi: 10.1371/journal.pntd.0014401 (PMC13271504; doi:10.1371/journal.pntd.0014401)
Supplement: S2 Table — Table list 9 animal models that have been developed for loiasis, the different stages used and the drugs used to validate the models. (PDF) [file pntd.0014401.s003.pdf]

Table 2. Summary of animal models for loiasis, worm stage used, key findings, limitations and drug use for validation

S/N	Model	Worm Stage	Key Findings	Limitations	Drug use for validation
1	*Papio anubis* (baboon)	L3 → mf	Reproduces ivermectin-associated adverse events; models hyper-microfilaraemia and neuropathology (82,83)	High cost; ethical constraints	IVM
2	CB.17 SCID mice	mf	Rapid clearance of mf following IVM; useful for microfilaricidal studies (84)	Limited immune function; partial parasite development	IVM
3	NOD SCID mice	L3	Limited support for parasite development; infection often cleared	Poor long-term maintenance	–
4	NOD.SCIDγc−/−	L3 → adult → mf	Supports full development to fertile adults and sustained infection (84,89)	Immune-deficient; lacks physiological	IVM

				immune response	
5	BALB/c RAG2−/−	L3	Partial parasite development; supports implantation models (84)	Limited immune context	IVM
6	BALB/c RAG2−/−γc−/−	L3 → adult → mf	Sustained parasite survival and microfilaraemia; suitable for long-term studies (84,89)	Immune-deficient	IVM
7	Cytokine-deficient mice (IL-4R, IL-5, IFN-γ, CCR-3 KO)	L3 → immature adult	Extended larval survival; highlights role of Th2 and eosinophils in parasite clearance (87–89)	Do not fully reproduce infection	–
8	WT BALB/c mice	mf	Transient mf presence; sequestration in cardiopulmonary system (84,87)	Short-lived infection	IVM
9	Gerbils	mf	Higher permissiveness than mice (64)	Mf localized in peritoneum	IVM

IVM= ivermectin, L3= third-stage larvae, Mf= microfilaria
