# Supplementary material for: Preclinical animal models for onchocerciasis and loiasis: A systematic review of applications in drug screening
Source: PLoS Negl Trop Dis. 2026 Jun 8;20(6):e0014401. doi: 10.1371/journal.pntd.0014401 (PMC13271504; doi:10.1371/journal.pntd.0014401)
Supplement: Attachment — Submitted filename: Answers to Reviewers comments.pdf [file pntd.0014401.s006.pdf]

Rebuttal Letter

Dr. Rene Bilingwe Ayiseh (Associate Professor)

Drugs and Molecular Diagnostic Laboratory (DMD),

Biotechnology Unit, University of Buea, Cameroon

Email: ayiseh.rene@ubuea.cm

Phone: +237 674 299 44 07

March 13, 2026

Dr. Maria Y. Pakharukova

Academic Editor

PLOS Neglected Tropical Diseases

plosntds@plos.org

Subject: Detailed Rebuttal to Reviewers' Comments for Manuscript (PNTD-D-25-01826) - [EMID:76449670819a5fb8]

Dear Dr. Pakharukova,

*We appreciate the opportunity to revise our manuscript, "**Preclinical Animal Models for Onchocerciasis and Loiasis: A Systematic Review of Applications in Drug Screening" (PNTD-D-25-01826) - [EMID:76449670819a5fb8],** based on the reviewers' feedback. We are grateful for their insightful comments which have significantly enhanced the manuscript's clarity, rigour, and overall impact. Below, we address each comment and outline the revisions made to the manuscript. We sincerely appreciate the time, expertise, and careful consideration the reviewers have invested in evaluating our manuscript. Their constructive feedback has been invaluable in strengthening both the scientific rigor and clarity of our work.*

Reviewer #1: This is a review which addressed an important subject about the control of debilitating disease in a co endemicity area of Central West Africa. It suggests animals' model which can be use to screen drugs for microfilaria or adult worm. a drug which can kill microfilaria and adult without any damage on human host is needed in this area. Therefore, the subject is important.

Thank you for your positive feedback on our manuscript. We are glad you find the subject important and relevant to controlling debilitating diseases in Central West Africa. We have incorporated the suggested corrections to improve the clarity of the manuscript. We are grateful for your encouraging assessment and for recognizing the relevance of this work in the context of co-endemic filarial diseases.

Reviewer #2: In this systematic review Ayiseh and colleagues attempt to coalesce publication data of numerous animal model systems of onchocerciasis and loiasis which have been applied for drug screening or immunopathology research.

I have the following major issues with the systematic review:

1. there is a lack of inclusion of many studies utilising the bovine ochengi natural infection system which has been utilised to test candidate macrofilaricides (tetracyclines, flubendazole, emodepside, moxidectin, long-acting ivermectin). Please see references of: Trees, Makepeace, Wanji. There should be a dedicated section reviewing these studies

Thank you for this important observation. We are particularly appreciative of this insightful comment, which helped us identify an important gap in the original manuscript. We agree that

the bovine Onchocerca ochengi natural infection model represents a cornerstone in onchocerciasis research, particularly for macrofilaricide development.

In response, we have now included a dedicated section titled "Natural and Large-Animal Models" (Lines 413–438). This section provides a comprehensive synthesis of studies utilising this model, including key contributions from Andrew J. Trees, Benjamin L. Makepeace, and Samuel Wanji.

We have expanded the discussion to include its application in evaluating macrofilaricidal candidates such as tetracyclines, flubendazole, ivermectin formulations, emodepside, and moxidectin, while also critically discussing its strengths and limitations relative to rodent models.

These additions significantly strengthen the translational relevance and completeness of the review.

Please find below what has been included in the manuscript

4.2.1 Natural and Large-Animal Models

The bovine *Onchocerca ochengi* natural infection model represents the most physiologically relevant and widely validated surrogate system for human onchocerciasis. As the closest phylogenetic relative of *O. volvulus*, *O. ochengi* shares similar transmission vectors, nodule formation, Wolbachia endosymbiosis, and drug susceptibility profiles, making it a cornerstone model for macrofilaricide discovery (Trees, Graham et al. 2000; Makepeace and Tanya 2016).

Over the past three decades, this model has been extensively utilised in both experimental and field settings, particularly in Cameroon, to evaluate candidate macrofilaricides and anti-Wolbachia therapies (Langworthy, Renz et al. 2000; Trees, Graham et al. 2000; Dec Bronsvoort, Makepeace et al. 2008; Makepeace and Tanya 2016), including recent translational drug candidates (Taylor, von Geldern et al. 2019). Notably, tetracycline-class antibiotics demonstrated potent macrofilaricidal activity through depletion of Wolbachia, leading to worm sterility and death(Hoerauf, Specht et al. 2008). Similarly, modified flubendazole formulations showed significant reductions in worm motility and viability, while long-acting ivermectin achieved sustained microfilarial clearance (Dec Bronsvoort, Makepeace et al. 2008; Boussinesq, Enyong et al. 2020). More recently, emodepside has emerged as a promising slow-acting macrofilaricide, inducing paralysis, sterility, and eventual death of adult worms (Bah et al., Plos Pathog, 2021).

A major strength of the bovine model lies in its ability to support the full parasite life cycle under natural infection conditions, enabling longitudinal assessment of drug efficacy on adult worms, nodules, and microfilariae. This provides a level of translational relevance that cannot be achieved in small animal models. However, despite its robustness, the model is constrained by high operational costs, logistical complexity, and ethical considerations associated with large animal use. These limitations have driven the development of complementary rodent models for early-stage drug screening. Nevertheless, the bovine *O. ochengi* system remains the gold standard for validating macrofilaricidal candidates prior to clinical development.

This model's unparalleled translational relevance is due to its close phylogenetic relationship with *O. volvulus*, shared vector biology, and validated responses to key drug classes

(Langworthy, Renz et al. 2000; Makepeace and Tanya 2016). Yet, its cost and complexity restrict routine use, underscoring the importance of scalable rodent models for initial screening.

We thank the reviewer for drawing our attention to this critical body of work, which has significantly enhanced the translational depth of the review.

2. the immunopathology section contains irrelevant references (e.g. human studies and many Litomosoides mouse studies which are out of context) and lacks many studies utilising onchocerca mf-specific mouse models of ocular keratitis and skin inflammation. Please see references of Pearlman and Bianco. The immunopathology section would need to be comprehensively re-written. I would alternatively recommend the systematic review simplified to drug screening models only and delete this section, as it is poorly developed.

After looking at the scope of the article and seeing that adding relevant studies on immunopathology will make the paper too lengthy and not very focused. We think your suggestion of completely removing the immunopathology section and focusing on drug screening models only is an excellent idea. We appreciate this thoughtful recommendation, which has helped us improve the focus, coherence, and readability of the manuscript. So, we have completely taken out the immunopathology study section of the manuscript.

3. The SCID mouse and gerbil male ochengi intraperitoneal implant models are not well described and overlooked in the summary table. These models where adult male worms are implanted and survive for 6-weeks have been used in a number of preclinical studies of anti-Wolbachia and direct

acting macrofilaricidal candidates (e.g. high dose rifampicin, ABBV-4083 (flubentylosin), quinazolines, flubendazole). These need to be included. See the work of Wanji, Turner.

Thank you for highlighting this oversight. We fully agree with the reviewer's assessment and are grateful for bringing this important omission to our attention. We agree that these models are crucial for preclinical screening. We have updated Table 1 to include the SCID mouse and gerbil Onchocerca ochengi male intraperitoneal implant models and have included a detailed description in the manuscript (Lines 251–256). The text below has been included in sections 3.1.2.1 (lines 202 - 220) and 3.1.4.1 (lines 250 - 269)

3.1.2.1 Intraperitoneal Adult Worm Implant Model in SCID Mice

A particularly important application of immunocompromised mouse models is the intraperitoneal implantation of adult male *O. ochengi* worms in SCID mice, which has been validated as a robust macrofilaricide screening platform (Halliday, Guimaraes et al. 2014; Sjoberg, Pionnier et al. 2019; Taylor, von Geldern et al. 2019; Akumtoh, Njouendou et al. 2021). In this model, recovered adult male worms from naturally infected cattle are implanted into the peritoneal cavity, where they remain viable and motile for approximately 5–6 weeks (Halliday, Guimaraes et al. 2014; Sjoberg, Pionnier et al. 2019). This survival window enables the assessment of drug-induced effects on worm motility, viability, and metabolic activity under controlled experimental conditions (Halliday, Guimaraes et al. 2014; Sjoberg, Pionnier et al. 2019; Akumtoh, Njouendou et al. 2021).

This SCID mouse implant model has been widely utilised in the preclinical evaluation of both anti-*Wolbachia* therapies and direct-acting macrofilaricidal compounds (Halliday, Guimaraes et al. 2014; Sjoberg, Pionnier et al. 2019; Taylor, von Geldern et al. 2019). Notably, high-dose rifamycin regimens (e.g. rifapentine/rifampicin-class) and next-

generation anti-Wolbachia candidates such as ABBV-4083 (flubentylosin) have demonstrated significant efficacy in depleting *Wolbachia* and impairing worm viability in filarial models, including *O. ochengi* implants and related systems (Halliday, Guimaraes et al. 2014; Taylor, von Geldern et al. 2019; Hegde, Marriott et al. 2024). In parallel, direct-acting compounds including flubendazole and related benzimidazoles or quinazoline/azaquinazoline derivatives have shown pronounced reductions in worm motility and survival in the O. ochengi SCID implant model and other rodent filariasis models (Halliday, Guimaraes et al. 2014; Sjoberg, Pionnier et al. 2019; Hubner, Martin et al. 2020; Hegde, Marriott et al. 2024). These studies, particularly those led by Wanji, Turner and collaborators, have established the SCID mouse male worm implant system as a reproducible and scalable model for macrofilaricide discovery.

3.1.4.1 Intraperitoneal Adult Male Implant Model in Gerbils

In addition to their role in general drug screening, gerbils have been extensively used in adult male *O. ochengi* intraperitoneal implant models, which complement SCID mouse systems and provide an alternative platform for macrofilaricide evaluation (Cho-Ngwa, Mbah et al. 2019; Mbah, Ayiseh et al. 2024). In this model, adult male worms are implanted into the peritoneal cavity of Mongolian gerbils, where they can survive for extended periods of up to 35–42 days with substantial recovery rates and preserved motility in untreated controls (Cho-Ngwa, Mbah et al. 2019; Mbah, Ayiseh et al. 2024).

The gerbil implant model has been particularly valuable in evaluating anti-*Wolbachia* therapies, including flubentylosin (ABBV-4083), where drug efficacy is assessed through reductions in *Wolbachia* load and subsequent impairment of worm viability (Hubner,

Koschel et al. 2019; von Geldern, Morton et al. 2019; Turner, Marriott et al. 2020; Johnston, Hong et al. 2021). Importantly, this model has also been used within broader rodent systems to assess pharmacokinetic–pharmacodynamic relationships across species, highlighting differences in drug exposure and efficacy between rodents(Hubner, Koschel et al. 2019; von Geldern, Morton et al. 2019; Turner, Marriott et al. 2020; Johnston, Hong et al. 2021). As such, the gerbil system provides complementary insights to SCID mouse models and strengthens confidence in translational drug candidates.

4. Wanji and McKenzie have latterly used the baboon NHP model of loiasis to investigate adverse reactions to ivermectin, these important studies should be included.

We thank the reviewer for highlighting these important studies, which substantially strengthen the safety and translational aspects of the review. The following text has been including in section 3.2.1 (lines 290 – 298).

Importantly, the baboon non-human primate (NHP) model of loiasis has been further refined by Wanji and McKenzie and colleagues, who demonstrated its utility in investigating ivermectin-associated adverse events (Wanji, Eyong et al. 2015; Wanji, Eyong et al. 2017; Pionnier, Sjoberg et al. 2019). In this model, hyper-microfilaraemic *Papio anubis* reproduced key clinical, hematological, and neuropathological features observed in humans following ivermectin treatment, including rapid microfilarial clearance and associated inflammatory responses (Wanji, Eyong et al. 2015; Herrick, Legrand et al. 2017; Wanji, Eyong et al. 2017). These studies have provided critical insights into the pathogenesis of severe adverse events and remain highly relevant for evaluating the safety of antifilarial interventions in *Loa loa*-

endemic settings (Wanji, Eyong et al. 2015; Wanji, Eyong et al. 2017; Pionnier, Sjoberg et al. 2019; Boulle, Pion et al. 2025).

minor comments:

We are grateful for these detailed and constructive minor comments, which have helped refine the precision and clarity of several sections of the manuscript.

210 - the humanised NSG infection model of Ov needs more careful description because several different humanisation strategies were tested. The authors also need to be careful to explain that although immature adults could be developed in these models, the worms were stunted and did not ever gain sexual maturity or form nodules.

Humanised NSG mouse models for Onchocerca volvulus aim to allow development of human-specific parasites in a small-animal host, but they currently only partially reproduce human infection. The following text has been added to section 3.2.1 (lines 192 – 201).

Humanised NSG mouse models of *O. volvulus* have employed distinct reconstitution strategies, including engraftment with human haematopoietic stem cells and/or immune or tissue components (CD34+ HSC "Hu-NSG", bone-marrow–liver–thymus "BLT" mice, and human skeletal muscle cell–engrafted NSG), which variably influence parasite development (Patton, Bennuru et al. 2018; Chen, Liao et al. 2022). While these models support progression from L3 to advanced L4 with development of male and female organs and an approximate 4-fold increase in larval length, parasite growth remains limited, with no evidence of fully mature adults, sexual reproduction or nodule formation in vivo (Patton,

Bennuru et al. 2018; Chen, Liao et al. 2022). These constraints highlight the partial recapitulation of human infection and the need for further optimisation of humanisation approaches and microenvironmental support to achieve complete development (Patton, Bennuru et al. 2018; Chen, Liao et al. 2022; Chunda, Fombad et al. 2024).

We appreciate the reviewer's careful attention to this nuance, which has improved the accuracy of our description.

215 - reference 51 details a SCID mouse male Oo implant model where Oo males survive for periods of 5-6 weeks and hence were validated as a macrofilaricide screen - this has not been detailed nor added to the summary table

This has now been detailed (see section 3.1.2.1; lines 202 - 220) and added to the summary table

3.1.2.1 Intraperitoneal Adult Worm Implant Model in SCID Mice

A particularly important application of immunocompromised mouse models is the intraperitoneal implantation of adult male *O. ochengi* worms in SCID mice, which has been validated as a robust macrofilaricide screening platform (Halliday, Guimaraes et al. 2014; Sjoberg, Pionnier et al. 2019; Taylor, von Geldern et al. 2019; Akumtoh, Njouendou et al. 2021). In this model, surgically recovered adult male worms from naturally infected cattle are implanted into the peritoneal cavity, where they remain viable and motile for approximately 5–6 weeks (Halliday, Guimaraes et al. 2014; Sjoberg, Pionnier et al. 2019). This survival window enables the assessment of drug-induced effects on worm motility, viability, and metabolic activity under controlled experimental conditions (Halliday, Guimaraes et al. 2014; Sjoberg, Pionnier et al. 2019; Akumtoh, Njouendou et al. 2021).

This SCID mouse implant model has been widely utilised in the preclinical evaluation of both anti-*Wolbachia* therapies and direct-acting macrofilaricidal compounds (Halliday, Guimaraes et al. 2014; Sjoberg, Pionnier et al. 2019; Taylor, von Geldern et al. 2019). Notably, high-dose rifamycin regimens (e.g. rifapentine/rifampicin-class) and next-generation anti-Wolbachia candidates such as ABBV-4083 (flubentylosin) have demonstrated significant efficacy in depleting *Wolbachia* and impairing worm viability in filarial models, including *O. ochengi* implants and related systems (Halliday, Guimaraes et al. 2014; Taylor, von Geldern et al. 2019; Hegde, Marriott et al. 2024). In parallel, direct-acting compounds including flubendazole and related benzimidazoles or quinazoline/azaquinazoline derivatives have shown pronounced reductions in worm motility and survival in the O. ochengi SCID implant model and other rodent filariasis models (Halliday, Guimaraes et al. 2014; Sjoberg, Pionnier et al. 2019; Hubner, Martin et al. 2020; Hegde, Marriott et al. 2024). These studies, particularly those led by Wanji, Turner and collaborators, have established the SCID mouse male worm implant system as a reproducible and scalable model for macrofilaricide discovery.

We thank the reviewer for noting this omission.

216 - the statement that immunocompromised mice are not ideal for screening drugs requiring the host immune response is without context or evidence and needs to be further justified and discussed. One could as easily argue that using semi-permissive rodent models (gerbils and hamsters for instance) where onchocerca are under immune-pressure, could also lead to artefactual macrofilaricidal evaluations.

This comment has helped us better contextualize the interpretation of model-dependent drug efficacy. This text below has been included in section 3.1.2.1 (lines 221-227).

Although immunocompromised mouse models are limited in evaluating drugs that rely on host immune-mediated mechanisms, they provide a controlled platform for assessing direct parasite-targeting effects, as exemplified by the SCID mouse implant model (Halliday, Guimaraes et al. 2014; Wiszniewsky, Layland et al. 2021; Chunda, Fombad et al. 2024). Conversely, semi-permissive immunocompetent models (e.g. gerbils and hamsters), where parasites are subject to host immune pressure, may introduce confounding effects that can overestimate macrofilaricidal efficacy (Cho-Ngwa, Mbah et al. 2019; Ayiseh, Mbah et al. 2023; Ayiseh, Mbah et al. 2023; Risch, Scheunemann et al. 2023). Thus, both systems present complementary advantages and limitations, and should be interpreted within the context of their immunological constraints.

248 - gerbil Oo male implants have been used in anti-Wolbachia drug screening (flubentylosin) - this is a good example of where it is useful to have more than one animal model when PK differs markedly between species.

This text below has been included in section 3.1.4.1 (lines 263-269).

Notably, the gerbil *O. ochengi* adult male implant model has also been applied in anti-Wolbachia drug screening, including flubentylosin (ABBV-4083), where reductions in Wolbachia burden correlate with impaired worm viability (Hubner, Koschel et al. 2019; Taylor, von Geldern et al. 2019; von Geldern, Morton et al. 2019). Importantly, comparative use of this model alongside SCID mouse systems has highlighted species-specific

pharmacokinetic differences that influence drug exposure and efficacy, underscoring the value of employing multiple complementary models in macrofilaricide development (Hubner, Koschel et al. 2019; Sjoberg, Pionnier et al. 2019; Taylor, von Geldern et al. 2019; Hubner, Martin et al. 2020).

We appreciate this valuable insight highlighting the importance of cross-species validation.

264 - table 1 - It would be useful to add a column to summarise the level of validation as a drug screen (types of compound etc) and references. As mentioned it is imperitive to include the cow model here as something of a gold standard.

The level of validation and drug used to validate the animal models have been added on Table 1 and the cow model has been included in the table as the gold standard.

We thank the reviewer for this helpful suggestion, which has improved the clarity and utility of the tables.

304 - it should be more fully explained that in mouse Loa mf models, peripheral mf in circulation are scant and that infused mf sequester in heart and lungs. Nonetheless IVM and other filaricides deplete mf from pulmonary circulation effectively, making this a validated system for testing microfilaricidal activity against Loa.

To this effect, the following text has been added in section 3.2.3 (lines 348-253).

In these mouse models, circulating *L. loa* microfilariae are typically scant and transient in peripheral blood, with the majority sequestered within the cardiopulmonary system, particularly the heart and lungs(Tendongfor, Wanji et al. 2012; Pionnier, Sjoberg et al. 2019). Despite this non-physiological distribution, ivermectin and other microfilaricidal

agents have consistently demonstrated effective clearance of microfilariae from these compartments, supporting the utility of this model for evaluating microfilaricidal activity against *L. loa* (Tendongfor, Wanji et al. 2012; Pionnier, Sjoberg et al. 2019; Ayiseh, Mbah et al. 2023).

330 - again 'the immune system' requirement of filaricidal compounds is without any justification or context.

The justification has now been included (section 3.1.2.1, lines 229-232)

Certain filaricidal interventions, particularly anti-Wolbachia therapies and immune-modulating compounds, rely in part on host immune effector mechanisms to achieve optimal efficacy (Voronin, Cook et al. 2012; Risch, Scheunemann et al. 2023; Fercoq, Cormerais et al. 2025). Consequently, immunocompromised models may underestimate the activity of such agents, as key immune pathways involved in parasite clearance are impaired (Halliday, Guimaraes et al. 2014; Risch, Scheunemann et al. 2023; Fercoq, Cormerais et al. 2025). This limitation should be considered when interpreting drug efficacy data generated from these systems.

358 - table 2 - please add references, types of compounds used for validation etc.

References and drug use to validate the animal models have been added on Table 2.

We thank the reviewer for this helpful suggestion, which has improved the clarity and utility of the tables.

441 - the discussion is overly long and repetitive. It should succinctly focus on pros- and cons- of current animal models of onchocerciasis and loiasis, and where further innovations might be required

We thank the reviewer for this important and constructive comment. We fully agree with the reviewer's assessment and have carefully revised the section to better align with the intended translational focus. In response, we have substantially revised and streamlined the Discussion section to reduce redundancy and improve clarity and focus. The revised Discussion now adopts a more structured and concise synthesis centered explicitly on the strengths and limitations of current animal models for onchocerciasis and loiasis, as well as key gaps requiring further innovation.

Specifically, we reorganized the section to (i) clearly delineate the advantages and constraints of major model systems, including the bovine Onchocerca ochengi natural infection model as the physiologically relevant gold standard, immunocompromised mouse models for controlled parasite development and drug screening, and semi-permissive rodent systems (gerbils and hamsters) for short- to medium-term evaluation of macrofilaricidal and microfilaricidal activity; and (ii) highlight critical limitations such as impaired immune competence in SCID/NSG models, non-physiological parasite localization in rodent systems (e.g., peritoneal or

cardiopulmonary sequestration), and the absence of a model that simultaneously supports full parasite development, physiological localization, and intact host immunity .

Furthermore, we have condensed repetitive descriptions and emphasized a forward-looking perspective, identifying priority areas for innovation. These include the development of refined humanized mouse models, improved co-infection platforms reflecting onchocerciasis–loiasis co-endemicity, and systems capable of integrating immune competence with accurate tissue-specific parasite biology. This revised synthesis aligns the Discussion more closely with the translational objective of identifying safe macrofilaricidal therapies while minimizing Loa loa–associated adverse events.

Overall, these revisions ensure that the Discussion is now more concise, non-redundant, and explicitly focused on translational strengths, limitations, and future directions, in line with the reviewer's recommendation. Find below how the discussion now reads.

4.0 DISCUSSION

4.1 Overview of Current Animal Models in a Translational Context

The present review synthesizes available animal models for onchocerciasis and loiasis within a translational framework centered on drug discovery in co-endemic settings. Across model systems, a recurring theme is that no single model fully recapitulates human infection, particularly in terms of parasite development, physiological localization, and host immune competence. Instead, each model captures specific aspects of parasite biology and drug response, necessitating a complementary, multi-model approach.

The bovine *Onchocerca ochengi* natural infection system remains the most physiologically relevant model, supporting the complete parasite life cycle under natural transmission

conditions and enabling longitudinal assessment of macrofilaricidal efficacy (Langworthy, Renz et al. 2000; Trees, Graham et al. 2000; Makepeace and Tanya 2016). However, its operational complexity and cost limit its use to late-stage validation. In contrast, small-animal models—particularly rodents—provide scalable platforms for early-stage screening, albeit with important biological constraints.

4.2 Strengths and Limitations of Onchocerciasis Models

4.2.1 Natural and Large-Animal Models

The bovine *Onchocerca ochengi* natural infection model represents the most physiologically relevant and widely validated surrogate system for human onchocerciasis. As the closest phylogenetic relative of *O. volvulus*, *O. ochengi* shares similar transmission vectors, nodule formation, Wolbachia endosymbiosis, and drug susceptibility profiles, making it a cornerstone model for macrofilaricide discovery (Trees, Graham et al. 2000; Makepeace and Tanya 2016).

Over the past three decades, this model has been extensively utilised in both experimental and field settings, particularly in Cameroon, to evaluate candidate macrofilaricides and anti-Wolbachia therapies (Langworthy, Renz et al. 2000; Trees, Graham et al. 2000; Dec Bronsvoort, Makepeace et al. 2008; Makepeace and Tanya 2016), including recent translational drug candidates (Taylor, von Geldern et al. 2019). Notably, tetracycline-class antibiotics demonstrated potent macrofilaricidal activity through depletion of Wolbachia, leading to worm sterility and death(Hoerauf, Specht et al. 2008). Similarly, modified flubendazole formulations showed significant reductions in worm motility and viability, while long-acting ivermectin achieved sustained microfilarial clearance (Dec Bronsvoort,

Makepeace et al. 2008; Boussinesq, Enyong et al. 2020). More recently, emodepside has emerged as a promising slow-acting macrofilaricide, inducing paralysis, sterility, and eventual death of adult worms (Bah et al., Plos Pathog, 2021).

A major strength of the bovine model lies in its ability to support the full parasite life cycle under natural infection conditions, enabling longitudinal assessment of drug efficacy on adult worms, nodules, and microfilariae. This provides a level of translational relevance that cannot be achieved in small animal models. However, despite its robustness, the model is constrained by high operational costs, logistical complexity, and ethical considerations associated with large animal use. These limitations have driven the development of complementary rodent models for early-stage drug screening. Nevertheless, the bovine *O. ochengi* system remains the gold standard for validating macrofilaricidal candidates prior to clinical development.

This model's unparalleled translational relevance is due to its close phylogenetic relationship with *O. volvulus*, shared vector biology, and validated responses to key drug classes (Langworthy, Renz et al. 2000; Makepeace and Tanya 2016). Yet, its cost and complexity restrict routine use, underscoring the importance of scalable rodent models for initial screening.

4.2.2 Immunocompromised Rodent Models

Immunodeficient mouse models (e.g., SCID, NSG) have significantly advanced the study of *O. volvulus* by permitting partial parasite development and supporting adult worm survival following implantation (Halliday, Guimaraes et al. 2014; Sjoberg, Pionnier et al. 2019). These systems provide highly controlled platforms for evaluating direct parasite-targeting drugs,

including anti-Wolbachia compounds such as flubentylosin and rifamycin derivatives (Taylor, von Geldern et al. 2019; Hegde, Marriott et al. 2024).

However, their major limitation is the absence of functional immune responses, which restricts their ability to evaluate therapies that rely on host immune mechanisms (Voronin, Cook et al. 2012; Risch, Scheunemann et al. 2023). Furthermore, humanized NSG models, although capable of supporting larval development to advanced stages, fail to achieve full parasite maturation, sexual reproduction, or nodule formation, reflecting only partial recapitulation of human infection (Patton, Bennuru et al. 2018; Chen, Liao et al. 2022; Chunda, Fombad et al. 2024).

4.2.3 Semi-Permissive Rodent Models (Gerbils and Hamsters)

Gerbils and hamsters provide practical and scalable models for drug screening, supporting short- to medium-term survival of *O. ochengi* microfilariae and adult worms (Mbah, Ayiseh et al. 2016; Cho-Ngwa, Mbah et al. 2019; Ayiseh, Mbah et al. 2023; Ayiseh, Mbah et al. 2023). Notably, adult male worm implant models in gerbils and SCID mice have been widely used for macrofilaricide evaluation, with reproducible worm recovery and viability over several weeks (Halliday, Guimaraes et al. 2014; Cho-Ngwa, Mbah et al. 2019; Sjoberg, Pionnier et al. 2019; Mbah, Ayiseh et al. 2024).

Despite these advantages, these models are constrained by non-physiological parasite localization, typically within the peritoneal cavity, and by variable parasite recovery rates. In addition, host immune pressure in immunocompetent rodents may introduce confounding effects that could overestimate drug efficacy (Ayiseh, Mbah et al. 2023; Risch, Scheunemann et al. 2023). These limitations underscore the need for cautious interpretation of efficacy data derived from such systems.

4.3 Strengths and Limitations of Loiasis Models

4.3.1 Non-Human Primate Models

Non-human primates (NHPs), particularly baboons, represent the most biologically accurate models of *Loa loa* infection, closely reproducing human microfilaraemia dynamics and clinical responses (Wanji, Eyong et al. 2015; Wanji, Eyong et al. 2017). These models have been critical in elucidating the pathogenesis of ivermectin-associated severe adverse events, including rapid microfilarial clearance and associated inflammatory responses.

However, their application is limited by ethical constraints, high costs, and restricted availability, rendering them unsuitable for routine drug screening.

4.3.2 Immunocompromised Mouse Models

Immunodeficient mouse models have emerged as valuable tools for studying *L. loa*, particularly for assessing microfilaricidal activity and adult worm development (Tendongfor, Wanji et al. 2012; Pionnier, Sjoberg et al. 2019). These models demonstrate that while peripheral microfilaraemia is often low and transient, microfilariae predominantly sequester in cardiopulmonary tissues, where they remain accessible to drug action.

Despite this non-physiological distribution, these systems have been validated for evaluating microfilaricidal efficacy, as demonstrated by consistent ivermectin-mediated clearance (Tendongfor, Wanji et al. 2012; Pionnier, Sjoberg et al. 2019). Nevertheless, similar to onchocerciasis models, their lack of immune competence limits their utility for studying immune-mediated mechanisms.

4.4 Translational Gaps and Limitations Across Models

A key finding from this review is the absence of a unified, scalable model that simultaneously satisfies three critical criteria: (i) physiological parasite localization, (ii) full parasite development and longevity, and (iii) intact host immune competence. While the bovine O. ochengi model closely fulfills these biological requirements—recapitulating natural parasite niches, sustained development, and host immune interactions—its limited scalability, high cost, and logistical complexity restrict its routine use in preclinical drug discovery pipelines. In contrast, immunocompromised rodent models enable parasite survival and partial development but lack the immune context necessary for evaluating host–parasite interactions and immunomodulatory therapies. Semi-permissive rodent models, although more practical and experimentally flexible, often compromise on physiological localization, reproducibility, and consistency of infection outcomes. Collectively, these limitations underscore a persistent trade-off between biological relevance and experimental tractability across existing filarial models.

4.5 Future Directions and Model Innovation

Addressing these gaps will require the development of next-generation integrative platforms. Humanized mouse models represent a promising direction, particularly if further refined to support: complete parasite maturation, physiological tissue localization and functional immune interactions dynamics.

Emerging in vitro approaches, including organoid systems and advanced co-culture models, are beginning to provide complementary platforms for filarial research (Duque-Correa, Maizels et al. 2019; Gandjui, Njouendou et al. 2021; Marriott, Furlong Silva et al.

2022). Although these systems remain underdeveloped for fully recapitulating parasite development and host–parasite interactions, recent studies highlight their potential for early-stage drug screening and mechanistic investigations (Voronin, Tricoche et al. 2019; Gandjui, Njouendou et al. 2021; Marriott, Furlong Silva et al. 2022; Risch, Scheunemann et al. 2023). Integration of such platforms with established animal models may enhance translational pipelines and reduce reliance on in vivo systems (Marriott, Furlong Silva et al. 2022; Risch, Scheunemann et al. 2023; Stresser, Kopec et al. 2023; Smith, Perez et al. 2025).

In addition, the development of co-infection models that can simultaneously accommodate *Onchocerca* and *Loa loa* parasites would significantly enhance the predictive value of preclinical studies, particularly for identifying macrofilaricidal compounds that avoid rapid *L. loa* microfilarial clearance.

Finally, a multi-model pipeline approach, integrating high-throughput rodent screening with validation in physiologically relevant systems such as the bovine model, may provide the most practical pathway for accelerating safe and effective drug discovery.

4.6 Conclusion

In summary, while substantial progress has been made in developing animal models for onchocerciasis and loiasis, each system presents distinct advantages and limitations. A strategic combination of these models, coupled with continued innovation in humanized and co-infection platforms, will be essential to overcome current translational barriers and to advance the development of safe macrofilaricidal therapies in co-endemic regions.

Reviewer #3: This manuscript presents an interesting and timely systematic review of preclinical animal models used in onchocerciasis and loiasis research, with clear relevance to drug screening and immunopathology, particularly in the context of co-endemicity and ivermectin-associated adverse events. The topic is important and the compilation of animal models across different host systems is valuable. However, some issues remain that limit the overall impact of the review. The structure of the manuscript would benefit from clearer organization and tighter alignment between objectives, results, and conclusions. In addition, the level of novelty is somewhat limited, as several of the described models and conclusions largely reiterate existing knowledge without sufficient critical synthesis or new conceptual insight. Finally, the review appears to lack inclusion and discussion of some relevant publications, which weakens the comprehensiveness expected of a systematic review. Addressing these issues would substantially strengthen the manuscript.

Detailed major concerns:

1) The overall narrative of the review is somewhat unclear. The authors introduce the filarial infections, then describe animal models, and subsequently return to immunological aspects, which are insufficiently developed. The section on immunology lacks depth and omits several key publications that specifically address immunological responses in animal models, including relevant work published by colleagues from Buea. In addition, an important recent study on

human filarial infections in mouse models is missing (Chunda et al., Frontiers in Tropical Diseases, 2024), which should be discussed to strengthen this aspect of the review.

After looking at the scope of the article and seeing that adding relevant studies on immunopathology will make the paper too lengthy and not very focused. We choose to go with one of the reviewers' suggestions to completely remove the immunopathology section and focus on drug screening models only.

The study by Chunda et al., Frontiers in Tropical Diseases, 2024 has now been incorporated to address this important omission and to strengthen the section on immunocompromised mouse models within the revised scope of the review. This study provides a comparative evaluation of the susceptibility of different immunocompromised mouse strains to human filarial infections, demonstrating that while Mansonella perstans and Onchocerca volvulus L3 failed to establish, Loa loa L3 achieved substantial recovery rates (62–66%) across strains, independent of sex, inoculation route, or immunosuppressive treatment. These findings highlight both the selective permissiveness and the limitations of current mouse models in recapitulating human filarial infections, while reinforcing their utility as controlled platforms for evaluating parasite establishment and drug effects. Accordingly, the study has been cited in the section on immunocompromised mouse models (Lines 302–340) to support a more balanced and up-to-date discussion of model performance and translational relevance.

2) The novelty of the review appears limited. A recent review focusing on animal models of Loa loa has already been published, and multiple reviews and primary studies on Onchocerca models are also available in the literature. The authors should more clearly articulate how the present

review advances beyond existing work, either through a new conceptual framework, comparative analysis, or deeper critical synthesis.

We sincerely thank the reviewer for this important and constructive comment. We appreciate the balanced and constructive nature of this critique, which has guided several important improvements in the manuscript. We have clarified the novelty of the manuscript in the revised Introduction, Methods, and Discussion sections.

The present review advances beyond existing literature in three principal ways:

1. ***Co-endemic translational framework***
   Unlike previous reviews that address Loa loa and Onchocerca models independently, this work integrates both systems within the context of co-endemicity and ivermectin-associated severe adverse events. This shifts the central question from model permissiveness to therapeutic selectivity, namely the identification of macrofilaricidal strategies effective against Onchocerca while minimizing risks linked to rapid L. loa microfilarial clearance.

2. ***Structured comparative synthesis of model systems***
   Rather than cataloguing models, we perform a side-by-side evaluation based on predefined translational criteria, including parasite stage support, survival duration, physiological localization, host immune competence, and suitability for immune-dependent versus independent drug action. This enables cross-parasite comparison within a unified preclinical framework.

3. ***Identification of translational gaps and future model directions***
   The review highlights a critical unmet need for models that simultaneously integrate

physiological relevance, immune competence, and sustained parasite survival. It further proposes a forward-looking roadmap, including humanized systems, co-infection platforms, and multi-model pipelines for safer macrofilaricide development.

These elements reposition the manuscript from a descriptive summary to a strategic, drug discovery–oriented synthesis tailored to co-endemic settings. Relevant clarifications have been incorporated into the Introduction, Methods, and Discussion (Sections 4.1 and 4.4–4.5).

There following has been added in the manuscript to fully explain the novelty of the review: introduction (lines 95 - 104), Method (lines 130 - 138) and Discussion (Sections 4.1 (lines 398-410) and 4.4–4.5 (lines 479 - 509)).

Introduction

Contrary to prior reviews that have examined either *L. loa* or *Onchocerca* animal models independently (Ngwewondo, Scandale et al. 2021; Wanji, Chunda et al. 2021), this review incorporates a translational co-endemic perspective. Given that MDA with IVM is constrained in areas where onchocerciasis and loiasis overlap due to severe adverse neurological reactions, preclinical model evaluation must move beyond parasite permissiveness (susceptibility) alone and instead address therapeutic selectivity and safety (Gardon, Gardon-Wendel et al. 1997; Twum-Danso and Meredith 2003; Cano, Basanez et al. 2018; Hawryluk, Zhiru et al. 2022).

We therefore comparatively synthesize animal models of both *Onchocerca* and *Loa* with emphasis on (i) their suitability for macrofilaricidal drug discovery, (ii) their relevance for identifying compounds effective against *Onchocerca* while avoiding rapid *L. loa* microfilaricidal effects, and (iii) their translational utility in co-endemic settings. This integrated perspective aims to bridge parasite biology with drug development to support the identification of safe and effective therapies.

Method

A structured comparative synthesis was conducted to enable cross-parasite evaluation of *Onchocerca* and *L. loa* systems within a unified preclinical development framework. This approach facilitated identification of complementary strengths and limitations across model systems, including differences in pharmacokinetic–pharmacodynamic profiles and host immune constraints. Emerging *in vitro* approaches, including organoid and co-culture systems, were identified during the screening process but were not included in the primary synthesis, as they remain insufficiently developed to recapitulate full parasite development and were outside the defined scope of preclinical animal models.

Discussion

4.1 Overview of Current Animal Models in a Translational Context

The present review synthesizes available animal models for onchocerciasis and loiasis within a translational framework centered on drug discovery in co-endemic settings. Across model systems, a recurring theme is that no single model fully recapitulates human infection, particularly in terms of parasite development, physiological localization, and host immune competence. Instead, each model captures specific aspects of parasite biology and drug response, necessitating a complementary, multi-model approach.

The bovine Onchocerca ochengi natural infection system remains the most physiologically relevant model, supporting the complete parasite life cycle under natural transmission conditions and enabling longitudinal assessment of macrofilaricidal efficacy (23,33,51). However, its operational complexity and cost limit its use to late-stage validation. In contrast, small-animal models—particularly rodents—provide scalable platforms for early-stage screening, albeit with important biological constraints.

4.4 Translational Gaps and Limitations Across Models

A key finding from this review is the absence of a unified, scalable model that simultaneously satisfies three critical criteria: (i) physiological parasite localization, (ii) full parasite development and longevity, and (iii) intact host immune competence. While the bovine O. ochengi model closely fulfills these biological requirements—recapitulating natural parasite niches, sustained development, and host immune interactions—its limited scalability, high cost, and logistical complexity restrict its routine use in preclinical drug discovery pipelines. In contrast, immunocompromised rodent models enable parasite survival and partial development but lack the immune context necessary for evaluating host–parasite interactions and immunomodulatory therapies. Semi-permissive rodent models, although more practical and experimentally flexible, often compromise on physiological localization, reproducibility, and consistency of infection outcomes. Collectively, these limitations underscore a persistent trade-off between biological relevance and experimental tractability across existing filarial models.

4.5 Future Directions and Model Innovation

Addressing these gaps will require the development of next-generation integrative platforms. Humanized mouse models represent a promising direction, particularly if further refined to support: complete parasite maturation, physiological tissue localization and functional immune interactions dynamics.

Emerging in vitro approaches, including organoid systems and advanced co-culture models, are beginning to provide complementary platforms for filarial research (96–98). Although these systems remain underdeveloped for fully recapitulating parasite development and host–parasite interactions, recent studies highlight their potential for early-stage drug screening and mechanistic investigations (95,96,98,99). Integration of such platforms with established animal models may enhance translational pipelines and reduce reliance on in vivo systems (95,96,100,101).

In addition, the development of co-infection models that can simultaneously accommodate *Onchocerca* and *Loa loa* parasites would significantly enhance the predictive value of preclinical

studies, particularly for identifying macrofilaricidal compounds that avoid rapid *L. loa* microfilarial clearance.

Finally, a multi-model pipeline approach, integrating high-throughput rodent screening with validation in physiologically relevant systems such as the bovine model, may provide the most practical pathway for accelerating safe and effective drug discovery.

We are grateful for this comment, which prompted us to more clearly articulate the unique contribution and positioning of our work within the existing literature.

3) The review does not address organoid systems or in vitro culture models. While these approaches are still under development for filarial research, recent publications demonstrate their growing potential for drug discovery and translational research. Including a discussion of these emerging methodologies would enhance the novelty, scope, and overall impact of the review.

The following has been included in the discussion (Section 4.5; lines 497- 502)

Emerging in vitro approaches, including organoid systems and advanced co-culture models, are beginning to provide complementary platforms for filarial research (Duque-Correa, Maizels et al. 2019; Gandjui, Njouendou et al. 2021; Marriott, Furlong Silva et al. 2022). Although these systems remain underdeveloped for fully recapitulating parasite development and host–parasite interactions, recent studies highlight their potential for early-stage drug screening and mechanistic investigations(Voronin, Tricoche et al. 2019; Gandjui, Njouendou et al. 2021; Marriott, Furlong Silva et al. 2022; Risch, Scheunemann et al. 2023). Integration of such platforms with established animal models may enhance translational pipelines and reduce reliance on in vivo systems (Marriott, Furlong Silva et al. 2022; Risch, Scheunemann et al. 2023; Stresser, Kopec et al. 2023; Smith, Perez et al. 2025).

We thank the reviewer for highlighting this emerging area, which has now been incorporated to broaden the scope and future perspective of the review.

We are sincerely grateful to the reviewers for their thoughtful, detailed, and constructive feedback. Their insights have significantly strengthened the quality, clarity, and translational relevance of this manuscript.

We hope that the revisions adequately address all concerns.

References

Akumtoh, D. N., A. J. Njouendou, et al. (2021). "Onchocerca ochengi male worms implanted in SCID mice and Gerbil: Relationship between microfilaridermia status of cows, nodular worm viability and fertility and worm survival in the rodents." Exp Parasitol 229: 108143.

Ayiseh, R. B., G. E. Mbah, et al. (2023). "Survival of worm masses of Onchocerca ochengi in gerbils and hamsters: implications for the development of an in vivo macrofilaricide screening model." Parasitology Research 122(7): 1581-1591.

Ayiseh, R. B., G. E. Mbah, et al. (2023). "Development and validation of small animal models for onchocerciasis and loiasis microfilaricide discovery." PLoS Negl Trop Dis 17(2): e0011135.

Boulle, C., S. D. Pion, et al. (2025). "Revisiting the Loa loa microfilaremia thresholds above which serious adverse events may occur with ivermectin treatment." PLoS Negl Trop Dis 19(3): e0012957.

Boussinesq, M., P. Enyong, et al. (2020). "Effects of an injectable long-acting formulation of ivermectin on Onchocerca ochengi in zebu cattle." Parasite (Paris, France) 27: 36.

Cano, J., M. G. Basanez, et al. (2018). "Identifying co-endemic areas for major filarial infections in sub-Saharan Africa: seeking synergies and preventing severe adverse events during mass drug administration campaigns." Parasit Vectors 11(1): 70.

Chen, J., S. Liao, et al. (2022). "The development and improvement of immunodeficient mice and humanized immune system mouse models." Front Immunol 13: 1007579.

Cho-Ngwa, F., G. E. Mbah, et al. (2019). "Development and validation of an Onchocerca ochengi adult male worm gerbil model for macrofilaricidal drug screening." PLoS Negl Trop Dis 13(7): e0007556.

Chunda, V. C., F. F. Fombad, et al. (2024). "Comparative development of human filariae Loa loa, Onchocerca volvulus and Mansonella perstans in immunocompromised mouse strains." Front Trop Dis 5: 1293632.

Dec Bronsvoort, B. M., B. L. Makepeace, et al. (2008). "UMF-078: A modified flubendazole with potent macrofilaricidal activity against Onchocerca ochengi in African cattle." Parasit Vectors 1(1): 18.

Duque-Correa, M. A., R. M. Maizels, et al. (2019). "Organoids - New Models for Host-Helminth Interactions." Trends Parasitol 36(2): 170-181.

Fercoq, F., C. Cormerais, et al. (2025). "Host environment shapes filarial parasite fitness and Wolbachia endosymbionts dynamics." PLoS Pathog 21(7): e1013301.

Gandjui, N. V. T., A. J. Njouendou, et al. (2021). "Establishment of an in vitro culture system to study the developmental biology of Onchocerca volvulus with implications for anti-Onchocerca drug discovery and screening." PLoS Negl Trop Dis 15(2): e0008513.

Gardon, J., N. Gardon-Wendel, et al. (1997). "Serious reactions after mass treatment of onchocerciasis with ivermectin in an area endemic for *Loa loa* infection." Lancet 350(9070): 18-22.

Halliday, A., A. Guimaraes, et al. (2014). "A murine macrofilaricide pre-clinical screening model for onchocerciasis and lymphatic filariasis." Parasites & Vectors 7(1): 1-14.

Hawryluk, N., L. Zhiru, et al. (2022). "Filarial nematode phenotypic screening cascade to identify compounds with anti-parasitic activity for drug discovery optimization." Int J Parasitol Drugs Drug Resist 19: 89-97.

Hegde, S., A. E. Marriott, et al. (2024). "Combinations of the azaquinazoline anti-Wolbachia agent, AWZ1066S, with benzimidazole anthelmintics synergise to mediate sub-seven-day sterilising and curative efficacies in experimental models of filariasis." Front Microbiol 15: 1346068.

Herrick, J. A., F. Legrand, et al. (2017). "Posttreatment Reactions After Single-Dose Diethylcarbamazine or Ivermectin in Subjects With Loa loa Infection." Clin Infect Dis 64(8): 1017-1025.

Hoerauf, A., S. Specht, et al. (2008). "Wolbachia endobacteria depletion by doxycycline as antifilarial therapy has macrofilaricidal activity in onchocerciasis: a randomized placebo-controlled study." Med Microbiol Immunol 197(3): 295-311.

Hubner, M. P., M. Koschel, et al. (2019). "In vivo kinetics of Wolbachia depletion by ABBV-4083 in L. sigmodontis adult worms and microfilariae." PLoS Negl Trop Dis 13(8): e0007636.

Hubner, M. P., C. Martin, et al. (2020). "Oxfendazole mediates macrofilaricidal efficacy against the filarial nematode Litomosoides sigmodontis in vivo and inhibits Onchocerca spec. motility in vitro." PLoS Negl Trop Dis 14(7): e0008427.

Johnston, K. L., W. D. Hong, et al. (2021). "Anti-Wolbachia drugs for filariasis." Trends Parasitol 37(12): 1068-1081.

Langworthy, N. G., A. Renz, et al. (2000). "Macrofilaricidal activity of tetracycline against the filarial nematode *Onchocerca ochengi*: elimination of *Wolbachia* precedes worm death and suggests a dependent relationship." Proc Biol Sci 267(1448): 1063-9.

Makepeace, B. L. and V. N. Tanya (2016). "25 Years of the Onchocerca ochengi Model." Trends in Parasitology 32(12): 966-978.

Marriott, A. E., J. Furlong Silva, et al. (2022). "A mouse infection model and long-term lymphatic endothelium co-culture system to evaluate drugs against adult Brugia malayi." PLoS Negl Trop Dis 16(6): e0010474.

Mbah, G. E., R. B. Ayiseh, et al. (2016). "Development and validation of an *Onchocerca ochengi* microfilarial hamster model for onchocerciasis drug screens." BMC Infect Dis 16(1): 404.

Mbah, G. E., R. B. Ayiseh, et al. (2024). "Differential susceptibility of Onchocerca ochengi adult male worms to flubendazole in gerbils and hamsters." Parasitology Research 123(4): 186.

Ngwewondo, A., I. Scandale, et al. (2021). "Onchocerciasis drug development: from preclinical models to humans." Parasitology Research 120(12): 3939-3964.

Patton, J. B., S. Bennuru, et al. (2018). "Development of Onchocerca volvulus in humanized NSG mice and detection of parasite biomarkers in urine and serum." PLOS Neglected Tropical Diseases 12(12): e0006977.

Pionnier, N. P., H. Sjoberg, et al. (2019). "Mouse models of Loa loa." Nature Communications 10(1): 1429.

Risch, F., J. F. Scheunemann, et al. (2023). "The efficacy of the benzimidazoles oxfendazole and flubendazole against Litomosoides sigmodontis is dependent on the adaptive and innate immune system." Front Microbiol 14: 1213143.

Sjoberg, H. T., N. Pionnier, et al. (2019). "Short-course, oral flubendazole does not mediate significant efficacy against Onchocerca adult male worms or Brugia microfilariae in murine infection models." PLoS Negl Trop Dis 13(1): e0006356.

Smith, D., M. G. Perez, et al. (2025). "Lessons on multicellular two- (2D) and three-dimensional (3D) culture in parasitology: Insights, challenges and future directions." Int J Parasitol: 104759.

Stresser, D. M., A. K. Kopec, et al. (2023). "Towards in vitro models for reducing or replacing the use of animals in drug testing." Nature Biomedical Engineering 8(8): 930-935.

Taylor, M. J., T. W. von Geldern, et al. (2019). "Preclinical development of an oral anti-Wolbachia macrolide drug for the treatment of lymphatic filariasis and onchocerciasis." Sci Transl Med 11(483).

Tendongfor, N., S. Wanji, et al. (2012). "The human parasite *Loa loa* in cytokine and cytokine receptor gene knock out BALB/c mice: survival, development and localization." Parasites & Vectors 5: 43-43.

Trees, A. J., S. P. Graham, et al. (2000). "*Onchocerca ochengi* infections in cattle as a model for human onchocerciasis: recent developments." Parasitology 120 Suppl: S133-42.

Turner, J. D., A. E. Marriott, et al. (2020). "Novel anti-Wolbachia drugs, a new approach in the treatment and prevention of veterinary filariasis?" Vet Parasitol 279: 109057.

Twum-Danso, N. A. and S. E. Meredith (2003). "Variation in incidence of serious adverse events after onchocerciasis treatment with ivermectin in areas of Cameroon co-endemic for loiasis." Trop Med Int Health 8(9): 820-31.

von Geldern, T. W., H. E. Morton, et al. (2019). "Discovery of ABBV-4083, a novel analog of Tylosin A that has potent anti-Wolbachia and anti-filarial activity." PLoS Negl Trop Dis 13(2): e0007159.

Voronin, D., D. A. Cook, et al. (2012). "Autophagy regulates Wolbachia populations across diverse symbiotic associations." Proc Natl Acad Sci U S A 109(25): E1638-46.

Voronin, D., N. Tricoche, et al. (2019). "Development of a preliminary in vitro drug screening assay based on a newly established culturing system for pre-adult fifth-stage Onchocerca volvulus worms." PLoS Negl Trop Dis 13(1): e0007108.

Wanji, S., V. C. Chunda, et al. (2021). "Advances in preclinical platforms of Loa loa for filarial neglected tropical disease drug and diagnostics research." Front Trop Dis 2.

Wanji, S., E.-E. Eyong, et al. (2015). "Parasitological, Hematological and Biochemical Characteristics of a Model of Hyper-microfilariaemic Loiasis (*Loa loa*) in the Baboon (*Papio anubis*)." PLoS Negl Trop Dis 9(11): e0004202.

Wanji, S., E. J. Eyong, et al. (2017). "Ivermectin treatment of Loa loa hyper-microfilaraemic baboons (Papio anubis): Assessment of microfilarial load reduction, haematological and biochemical parameters and histopathological changes following treatment." PLoS Negl Trop Dis 11(7): e0005576.

Wiszniewsky, A., L. E. Layland, et al. (2021). "Adoptive Transfer of Immune Cells Into RAG2IL-2Rgamma-Deficient Mice During Litomosoides sigmodontis Infection: A Novel Approach to Investigate Filarial-Specific Immune Responses." Front Immunol 12: 777860.
