# Supplementary material for: Preclinical animal models for onchocerciasis and loiasis: A systematic review of applications in drug screening
Source: PLoS Negl Trop Dis. 2026 Jun 8;20(6):e0014401. doi: 10.1371/journal.pntd.0014401 (PMC13271504; doi:10.1371/journal.pntd.0014401)
Supplement: Peer review history

# Peer review: Decision Letter 0

**DOI:** 10.1371/journal.pntd.0014401.r001

**Authors:** Maria Pakharukova

19 Jan 2026

-->PNTD-D-25-01826

Preclinical Animal Models for Onchocerciasis and Loiasis: A Systematic Review of Applications in Drug Screening and Immunopathology

PLOS Neglected Tropical Diseases

Dear Dr. Ayiseh,

Thank you for submitting your manuscript to PLOS Neglected Tropical Diseases. After careful consideration, we feel that it has merit but does not fully meet PLOS Neglected Tropical Diseases's publication criteria as it currently stands. Therefore, we invite you to submit a revised version of the manuscript that addresses the points raised during the review process.

Please submit your revised manuscript within by Mar 20 2026 11:59PM. If you will need more time than this to complete your revisions, please reply to this message or contact the journal office at plosntds@plos.org. When you're ready to submit your revision, log on to https://www.editorialmanager.com/pntd/ and select the 'Submissions Needing Revision' folder to locate your manuscript file.

Please include the following items when submitting your revised manuscript:

* A letter that responds to each point raised by the editor and reviewer(s). You should upload this letter as a separate file labeled 'Response to Reviewers'. This file does not need to include responses to any formatting updates and technical items listed in the 'Journal Requirements' section below.

* A marked-up copy of your manuscript that highlights changes made to the original version. You should upload this as a separate file labeled 'Revised Manuscript with Track Changes'.

* An unmarked version of your revised paper without tracked changes. You should upload this as a separate file labeled 'Manuscript'.

If you would like to make changes to your financial disclosure, competing interests statement, or data availability statement, please make these updates within the submission form at the time of resubmission. Guidelines for resubmitting your figure files are available below the reviewer comments at the end of this letter.

We look forward to receiving your revised manuscript.

Kind regards,

Maria Y Pakharukova, Ph.D., D.Sc.

Academic Editor

PLOS Neglected Tropical Diseases

Jong-Yil Chai

Section Editor

PLOS Neglected Tropical Diseases

Shaden Kamhawi

co-Editor-in-Chief

PLOS Neglected Tropical Diseases

orcid.org/0000-0003-4304-636XX

Paul Brindley

co-Editor-in-Chief

PLOS Neglected Tropical Diseases

orcid.org/0000-0003-1765-0002

**Journal Requirements:**

If the reviewer comments include a recommendation to cite specific previously published works, please review and evaluate these publications to determine whether they are relevant and should be cited. There is no requirement to cite these works unless the editor has indicated otherwise.

1) Please upload all main figures as separate Figure files in .tif or .eps format. For more information about how to convert and format your figure files please see our guidelines:

https://journals.plos.org/plosntds/s/figures

2) We have noticed that you have uploaded Supporting Information files, but you have not included a list of legends. Please add a full list of legends for your Supporting Information files after the references list.

3) We note that your Data Availability Statement is currently as follows: "Not applicable - this is a systematic review". Please confirm at this time whether or not your submission contains all raw data required to replicate the results of your study. Authors must share the “minimal data set” for their submission. PLOS defines the minimal data set to consist of the data required to replicate all study findings reported in the article, as well as related metadata and methods (https://journals.plos.org/plosone/s/data-availability#loc-minimal-data-set-definition).

For example, authors should submit the following data:

1) The values behind the means, standard deviations and other measures reported;

2) The values used to build graphs;

3) The points extracted from images for analysis..

Authors do not need to submit their entire data set if only a portion of the data was used in the reported study.

If your submission does not contain these data, please either upload them as Supporting Information files or deposit them to a stable, public repository and provide us with the relevant URLs, DOIs, or accession numbers. For a list of recommended repositories, please see https://journals.plos.org/plosone/s/recommended-repositories.

If there are ethical or legal restrictions on sharing a de-identified data set, please explain them in detail (e.g., data contain potentially sensitive information, data are owned by a third-party organization, etc.) and who has imposed them (e.g., an ethics committee). Please also provide contact information for a data access committee, ethics committee, or other institutional body to which data requests may be sent. If data are owned by a third party, please indicate how others may request data access.

4) As required by our policy on Data Availability, please ensure your manuscript or supplementary information includes the following:

- A numbered table of all studies identified in the literature search, including those that were excluded from the analyses.

- For every excluded study, the table should list the reason(s) for exclusion.

- If any of the included studies are unpublished, include a link (URL) to the primary source or detailed information about how the content can be accessed.

- A table of all data extracted from the primary research sources for the systematic review and/or meta-analysis. The table must include the following information for each study:

- Name of data extractors and date of data extraction

- Confirmation that the study was eligible to be included in the review.

- All data extracted from each study for the reported systematic review and/or meta-analysis that would be needed to replicate your analyses.

- If data or supporting information were obtained from another source (e.g. correspondence with the author of the original research article), please provide the source of data and dates on which the data/information were obtained by your research group.

- If applicable for your analysis, a table showing the completed risk of bias and quality/certainty assessments for each study or outcome.  Please ensure this is provided for each domain or parameter assessed. For example, if you used the Cochrane risk-of-bias tool for randomized trials, provide answers to each of the signalling questions for each study. If you used GRADE to assess certainty of evidence, provide judgements about each of the quality of evidence factor. This should be provided for each outcome.

- An explanation of how missing data were handled.

This information can be included in the main text, supplementary information, or relevant data repository. Please note that providing these underlying data is a requirement for publication in this journal, and if these data are not provided your manuscript might be rejected.

**Reviewers' Comments:**

Reviewer's Responses to Questions

**Key Review Criteria Required for Acceptance?**

As you describe the new analyses required for acceptance, please consider the following:

**Methods**

-Are the objectives of the study clearly articulated with a clear testable hypothesis stated?

-Is the study design appropriate to address the stated objectives?

-Is the population clearly described and appropriate for the hypothesis being tested?

-Is the sample size sufficient to ensure adequate power to address the hypothesis being tested?

-Were correct statistical analysis used to support conclusions?

-Are there concerns about ethical or regulatory requirements being met?

Reviewer #1: This juste a review based on meta analysi. tha objectives of the study is clearly articulate. The study design is correct. Others considerations are not appropriate in this specific case.

Reviewer #2: objectives are clearly articulated but in this systematic review, a number of omissions of the relevant literature are apparent (see below)

Reviewer #3: (No Response)

**********

**Results**

-Does the analysis presented match the analysis plan?

-Are the results clearly and completely presented?

-Are the figures (Tables, Images) of sufficient quality for clarity?

Reviewer #1: The analysis matched the analysis plan, the results clearly and completely presented

Reviewer #2: yes

Reviewer #3: (No Response)

**********

**Conclusions**

-Are the conclusions supported by the data presented?

-Are the limitations of analysis clearly described?

-Do the authors discuss how these data can be helpful to advance our understanding of the topic under study?

-Is public health relevance addressed?

Reviewer #1: The conclusion is supported by the data presented, the limitations of the analysis presented, and the authors discuss how these data can be helpful to advance our understanding of the topic under study, and the public health is addressed.

Reviewer #2: partially, there are many references lacking on the immunopathology aspects of onchocerciasis utilsing mouse models.

Reviewer #3: (No Response)

**********

**Editorial and Data Presentation Modifications?**

Use this section for editorial suggestions as well as relatively minor modifications of existing data that would enhance clarity. If the only modifications needed are minor and/or editorial, you may wish to recommend “Minor Revision” or “Accept”.

Reviewer #1: No

Reviewer #2: some attention to language is needed (see below)

Reviewer #3: (No Response)

**********

**Summary and General Comments**

Use this section to provide overall comments, discuss strengths/weaknesses of the study, novelty, significance, general execution and scholarship. You may also include additional comments for the author, including concerns about dual publication, research ethics, or publication ethics. If requesting major revision, please articulate the new experiments that are needed.

Reviewer #1: This is a review which addressed an important subject about the control of debilitating disease in a co endemicity area of Central West Africa. It suggest animals model which can be use to screen drugs for microfilaria or adult worm. a drug which can kill microfilaria and and adult whithout any damage on human host is needed in this area. Therefore the subject is important .

Reviewer #2: In this systematic review Ayiseh and colleagues attempt to coalesce publication data of numerous animal model systems of onchocerciasis and loiasis which have been applied for drug screening or immunopathology research.

I have the following major issues with the systematic review:

1. there is a lack of inclusion of many studies utilising the bovine ochengi natural infection system which has been utilised to test candidate macrofilaricides (tetracyclines, flubendazole, emodepside, moxidectin, long-acting ivermectin). Please see references of: Trees, Makepeace, Wanji. There should be a dedicated section reviewing these studies

2. the immunopathology section contains irrelevant references (e.g. human studies and many Litomosoides mouse studies which are out of context) and lacks many studies utilising onchocerca mf-specific mouse models of ocular keratitis and skin inflammation. Please see references of Pearlman and Bianco. The immunopathology section would need to be comprehensively re-written. I would alternatively recommend the systematic review simplified to drug screening models only and delete this section, as it is poorly developed.

3. The SCID mouse and gerbil male ochengi intraperitoneal implant models are not well described and overlooked in the summary table. These models where adult male worms are implanted and survive for 6-weeks have been used in a number of preclinical studies of anti-Wolbachia and direct acting macrofilaricidal candidates (e.g. high dose rifampicin, ABBV-4083 (flubentylosin), quinazolines, flubendazole). These need to be included. See the work of Wanji, Turner.

4. Wanji and McKenzie have latterly used the baboon NHP model of loiasis to investigate adverse reactions to ivermectin, these important studies should be included.

minor comments:

210 - the humanised NSG infection model of Ov needs more careful description because several different humanisation strategies were tested. The authors also need to be careful to explain that although immature adults could be developed in these models, the worms were stunted and did not ever gain sexual maturity or form nodules.

215 - reference 51 details a SCID mouse male Oo implant model where Oo males survive for periods of 5-6 weeks and hence were validated as a macrofilaricide screen - this has not been detailed nor added to the summary table

216 - the statement that immunocompromised mice are not ideal for screening drugs requiring the host immune response is without context or evidence and needs to be further justified and discussed. One could as easily argue that using semi-permissive rodent models (gerbils and hamsters for instance) where onchocerca are under immune-pressure, could also lead to artefactual macrofilaricidal evaluations.

248 - gerbil Oo male implants have been used in anti-Wolbachia drug screening (flubentylosin) - this is a good example of where it is useful to have more than one animal model when PK differs markedly between species.

264 - table 1 - It would be useful to add a column to summarise the level of validation as a drug screen (types of compound etc) and references. As mentioned it is imperitive to include the cow model here as something of a gold standard.

304 - it should be more fully explained that in mouse Loa mf models, peripheral mf in circulation are scant and that infused mf sequester in heart and lungs. Nonetheless IVM and other filaricides deplete mf from pulmonary circulation effectively, making this a validated system for testing microfilaricidal activity against Loa.

330 - again 'the immune system' requirement of filaricidal compounds is without any justification or context.

358 - table 2 - please add references, types of compounds used for validation etc.

441 - the discussion is overly long and repetitive. It should succinctly focus on pros- and cons- of current animal models of onchocerciasis and loiasis, and where further innovations might be required

Reviewer #3: This manuscript presents an interesting and timely systematic review of preclinical animal models used in onchocerciasis and loiasis research, with clear relevance to drug screening and immunopathology, particularly in the context of co-endemicity and ivermectin-associated adverse events. The topic is important and the compilation of animal models across different host systems is valuable. However, some issues remain that limit the overall impact of the review. The structure of the manuscript would benefit from clearer organization and tighter alignment between objectives, results, and conclusions. In addition, the level of novelty is somewhat limited, as several of the described models and conclusions largely reiterate existing knowledge without sufficient critical synthesis or new conceptual insight. Finally, the review appears to lack inclusion and discussion of some relevant publications, which weakens the comprehensiveness expected of a systematic review. Addressing these issues would substantially strengthen the manuscript.

Detailed major concerns:

1) The overall narrative of the review is somewhat unclear. The authors introduce the filarial infections, then describe animal models, and subsequently return to immunological aspects, which are insufficiently developed. The section on immunology lacks depth and omits several key publications that specifically address immunological responses in animal models, including relevant work published by colleagues from Buea. In addition, an important recent study on human filarial infections in mouse models is missing (Chunda et al., Frontiers in Tropical Diseases, 2024), which should be discussed to strengthen this aspect of the review.

2) The novelty of the review appears limited. A recent review focusing on animal models of Loa loa has already been published, and multiple reviews and primary studies on Onchocerca models are also available in the literature. The authors should more clearly articulate how the present review advances beyond existing work, either through a new conceptual framework, comparative analysis, or deeper critical synthesis.

3) The review does not address organoid systems or in vitro culture models. While these approaches are still under development for filarial research, recent publications demonstrate their growing potential for drug discovery and translational research. Including a discussion of these emerging methodologies would enhance the novelty, scope, and overall impact of the review.

**********

PLOS authors have the option to publish the peer review history of their article (what does this mean?). If published, this will include your full peer review and any attached files.

If you choose “no”, your identity will remain anonymous but your review may still be made public.

**Do you want your identity to be public for this peer review?**  For information about this choice, including consent withdrawal, please see our Privacy Policy.

Reviewer #1: No

Reviewer #2: No

Reviewer #3: No

**Figure resubmission:**

While revising your submission, we strongly recommend that you use PLOS’s NAAS tool (https://ngplosjournals.pagemajik.ai/artanalysis) to test your figure files. NAAS can convert your figure files to the TIFF file type and meet basic requirements (such as print size, resolution), or provide you with a report on issues that do not meet our requirements and that NAAS cannot fix.

-->After uploading your figures to PLOS’s NAAS tool - https://ngplosjournals.pagemajik.ai/artanalysis, NAAS will process the files provided and display the results in the "Uploaded Files" section of the page as the processing is complete. If the uploaded figures meet our requirements (or NAAS is able to fix the files to meet our requirements), the figure will be marked as "fixed" above. If NAAS is unable to fix the files, a red "failed" label will appear above. When NAAS has confirmed that the figure files meet our requirements, please download the file via the download option, and include these NAAS processed figure files when submitting your revised manuscript.-->

**Reproducibility:**

To enhance the reproducibility of your results, we recommend that authors of applicable studies deposit laboratory protocols in protocols.io, where a protocol can be assigned its own identifier (DOI) such that it can be cited independently in the future. Additionally, PLOS ONE offers an option to publish peer-reviewed clinical study protocols. Read more information on sharing protocols at https://plos.org/protocols?utm_medium=editorial-email&utm_source=authorletters&utm_campaign=protocols

-->

# Author comment: Author response to Decision Letter 1

**DOI:** 10.1371/journal.pntd.0014401.r002

24 Apr 2026

Attachment Submitted filename: Answers to Reviewers comments.pdf

# Peer review: Decision Letter 1

**DOI:** 10.1371/journal.pntd.0014401.r003

**Authors:** Maria Pakharukova

21 May 2026

Dear Dr Ayiseh,

We are pleased to inform you that your manuscript 'Preclinical Animal Models for Onchocerciasis and Loiasis: A Systematic Review of Applications in Drug Screening' has been provisionally accepted for publication in PLOS Neglected Tropical Diseases.

Before your manuscript can be formally accepted you will need to complete some formatting changes, which you will receive in a follow up email. A member of our team will be in touch with a set of requests.

Please note that your manuscript will not be scheduled for publication until you have made the required changes, so a swift response is appreciated.

IMPORTANT: The editorial review process is now complete. PLOS will only permit corrections to spelling, formatting or significant scientific errors from this point onwards. Requests for major changes, or any which affect the scientific understanding of your work, will cause delays to the publication date of your manuscript.

Should you, your institution's press office or the journal office choose to press release your paper, you will automatically be opted out of early publication. We ask that you notify us now if you or your institution is planning to press release the article. All press must be co-ordinated with PLOS.

Thank you again for supporting Open Access publishing; we are looking forward to publishing your work in PLOS Neglected Tropical Diseases.

Best regards,

Maria Y Pakharukova, Ph.D., D.Sc.

Academic Editor

PLOS Neglected Tropical Diseases

Jong-Yil Chai

Section Editor

PLOS Neglected Tropical Diseases

Shaden Kamhawi

co-Editor-in-Chief

PLOS Neglected Tropical Diseases

orcid.org/0000-0003-4304-636XX

Paul Brindley

co-Editor-in-Chief

PLOS Neglected Tropical Diseases

orcid.org/0000-0003-1765-0002

***********************************************************

p.p1 {margin: 0.0px 0.0px 0.0px 0.0px; line-height: 16.0px; font: 14.0px Arial; color: #323333; -webkit-text-stroke: #323333}span.s1 {font-kerning: none

Reviewer's Responses to Questions

**Key Review Criteria Required for Acceptance?**

As you describe the new analyses required for acceptance, please consider the following:

**Methods**

-Are the objectives of the study clearly articulated with a clear testable hypothesis stated?

-Is the study design appropriate to address the stated objectives?

-Is the population clearly described and appropriate for the hypothesis being tested?

-Is the sample size sufficient to ensure adequate power to address the hypothesis being tested?

-Were correct statistical analysis used to support conclusions?

-Are there concerns about ethical or regulatory requirements being met?

Reviewer #2: this is a systematic review

Reviewer #3: (No Response)

**********

**Results**

-Does the analysis presented match the analysis plan?

-Are the results clearly and completely presented?

-Are the figures (Tables, Images) of sufficient quality for clarity?

Reviewer #2: yes

Reviewer #3: (No Response)

**********

**Conclusions**

-Are the conclusions supported by the data presented?

-Are the limitations of analysis clearly described?

-Do the authors discuss how these data can be helpful to advance our understanding of the topic under study?

-Is public health relevance addressed?

Reviewer #2: yes

Reviewer #3: (No Response)

**********

**Editorial and Data Presentation Modifications?**

Use this section for editorial suggestions as well as relatively minor modifications of existing data that would enhance clarity. If the only modifications needed are minor and/or editorial, you may wish to recommend “Minor Revision” or “Accept”.

Reviewer #2: none

Reviewer #3: (No Response)

**********

**Summary and General Comments**

Use this section to provide overall comments, discuss strengths/weaknesses of the study, novelty, significance, general execution and scholarship. You may also include additional comments for the author, including concerns about dual publication, research ethics, or publication ethics. If requesting major revision, please articulate the new experiments that are needed.

Reviewer #2: thanks to the authors for narrowing the scope of the review and improving accuracy and details addressing my review. I have no further comments and suggest the review is acceptable for publication.

Reviewer #3: (No Response)

**********

PLOS authors have the option to publish the peer review history of their article (what does this mean?). If published, this will include your full peer review and any attached files.

If you choose “no”, your identity will remain anonymous but your review may still be made public.

**Do you want your identity to be public for this peer review?**  For information about this choice, including consent withdrawal, please see our Privacy Policy.

Reviewer #2: No

Reviewer #3: No

# Editor report: Acceptance letter

**DOI:** 10.1371/journal.pntd.0014401.r004

**Authors:** Maria Pakharukova

Dear Dr Ayiseh,

We are delighted to inform you that your manuscript, "Preclinical Animal Models for Onchocerciasis and Loiasis: A Systematic Review of Applications in Drug Screening," has been formally accepted for publication in PLOS Neglected Tropical Diseases.

We have now passed your article onto the PLOS Production Department who will complete the rest of the publication process. All authors will receive a confirmation email upon publication.

The corresponding author will soon be receiving a typeset proof for review, to ensure errors have not been introduced during production. Please review the PDF proof of your manuscript carefully, as this is the last chance to correct any scientific or type-setting errors. Please note that major changes, or those which affect the scientific understanding of the work, will likely cause delays to the publication date of your manuscript. Note: Proofs for Front Matter articles (Editorial, Viewpoint, Symposium, Review, etc...) are generated on a different schedule and may not be made available as quickly.

Soon after your final files are uploaded, the early version of your manuscript will be published online unless you opted out of this process. The date of the early version will be your article's publication date. The final article will be published to the same URL, and all versions of the paper will be accessible to readers.

For Research Articles, you will receive an invoice from PLOS for your publication fee after your manuscript has reached the completed accept phase. If you receive an email requesting payment before acceptance or for any other service, this may be a phishing scheme. Learn how to identify phishing emails and protect your accounts at https://explore.plos.org/phishing.

Thank you again for supporting open-access publishing; we are looking forward to publishing your work in PLOS Neglected Tropical Diseases.

Best regards,

Shaden Kamhawi

co-Editor-in-Chief

PLOS Neglected Tropical Diseases

Paul Brindley

co-Editor-in-Chief

PLOS Neglected Tropical Diseases
